# Supplementary material for: Unmasking complex kinetics in viral entry by inferring hypoexponential models
Source: Biophys J. 2025 Oct 25;124(23):4356–67. doi: 10.1016/j.bpj.2025.10.035 (PMC12709397; doi:10.1016/j.bpj.2025.10.035)
Supplement: Document S1. Figures S1–S8 [file mmc1.pdf]

Biophysical Journal, Volume *124*

Supplemental information

Unmasking complex kinetics in viral entry by inferring hypoexponential models

Oyinkansola Adenekan and Peter M. Kasson

Supplementary Information for

Unmasking complex kinetics in viral entry by inferring hypoexponential models

Oyinkansola Adenekan[1] and Peter M. Kasson[1,2,3*]

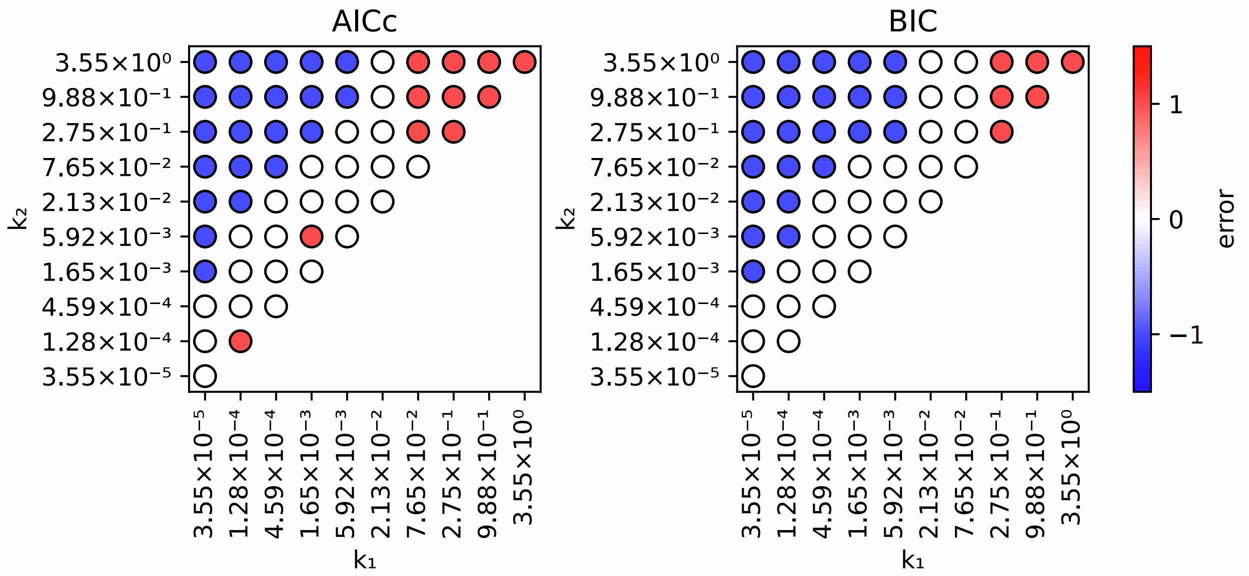

Figure S1. Model selection using AICc versus Bayesian Information Criterion (BIC). The estimated number of steps N was selected using AICc or BIC on hypoexponential sampling of models with 1-5 steps. This was performed on three independent sampling runs, and the majority-vote selection was adopted. This is analogous to the AICc model selection presented in Figure 3c.

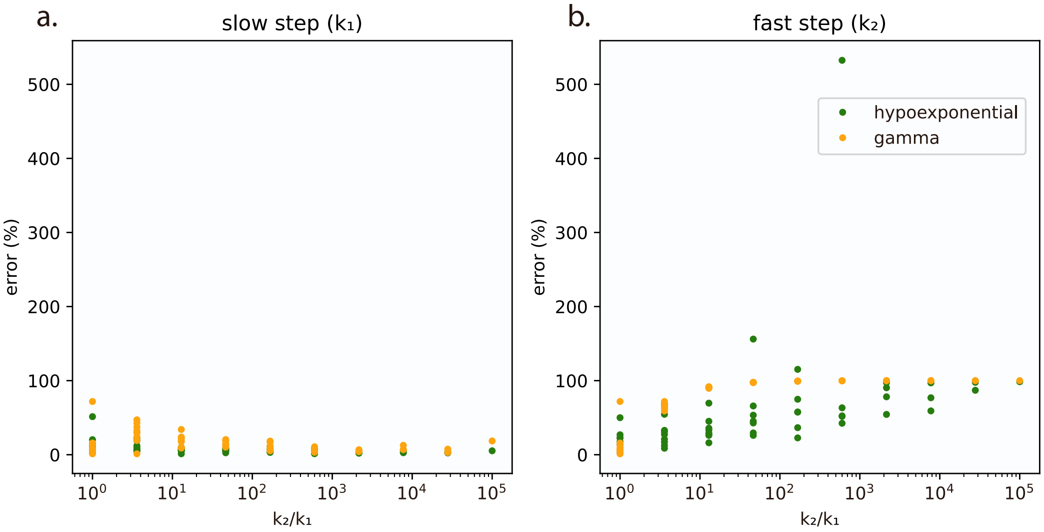

Figure S2. Rate errors as a function of k_2/k_1 ratio. Data from Figure 3 are replotted as a function of k_2/k_1 ratio in panel (a) for k_1 and panel (b) for k_2.

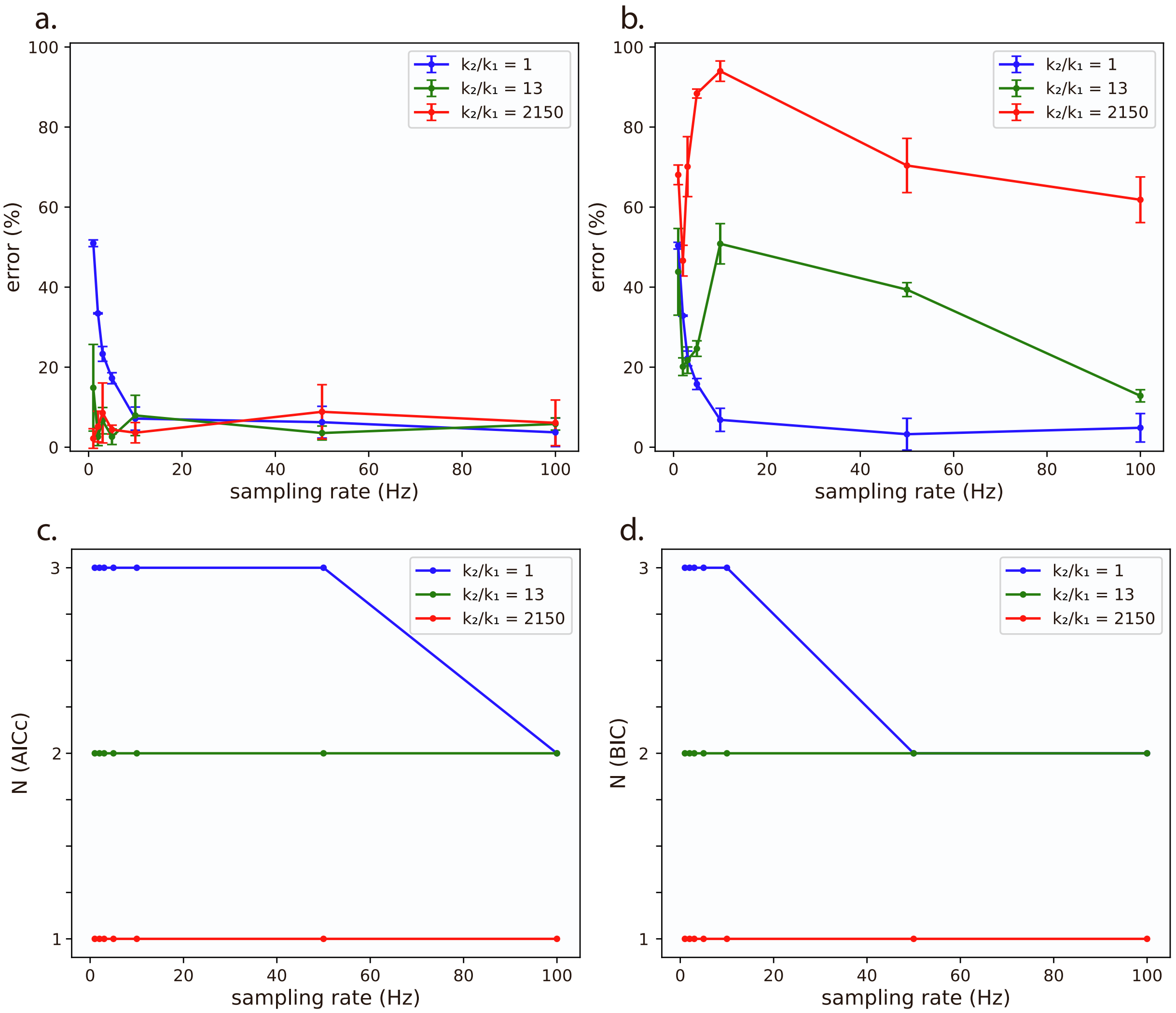

Figure S3. Dependence of error on sampling rate. Synthetic data were generated to emulate experimental sampling rates or time resolutions varying from 1 Hz to 100 Hz, and the error was calculated for three different two-step rate regimes, k_1 = 3.55 s^{-1} and k_2 = 3.55 s^{-1} (blue), k_1 = 2.13e-02 s^{-1} and k_2 = 2.75e-01 s^{-1} (green), and k_1 = 4.59e-04 s^{-1} and k2 = 9.88e-01 s^{-1} (red). The errors were plotted in panel (a) for k_1, panel (b) for k_2, panel (c) for N using AICc model selection, and panel (d) for N using BIC model selection. In all cases, increasing the sampling rate reduced error for faster rates. Three separate runs of hypoexponential sampling were performed, and error bars in panels a-b show standard deviation. Panels c and d show errors based on majority-vote.

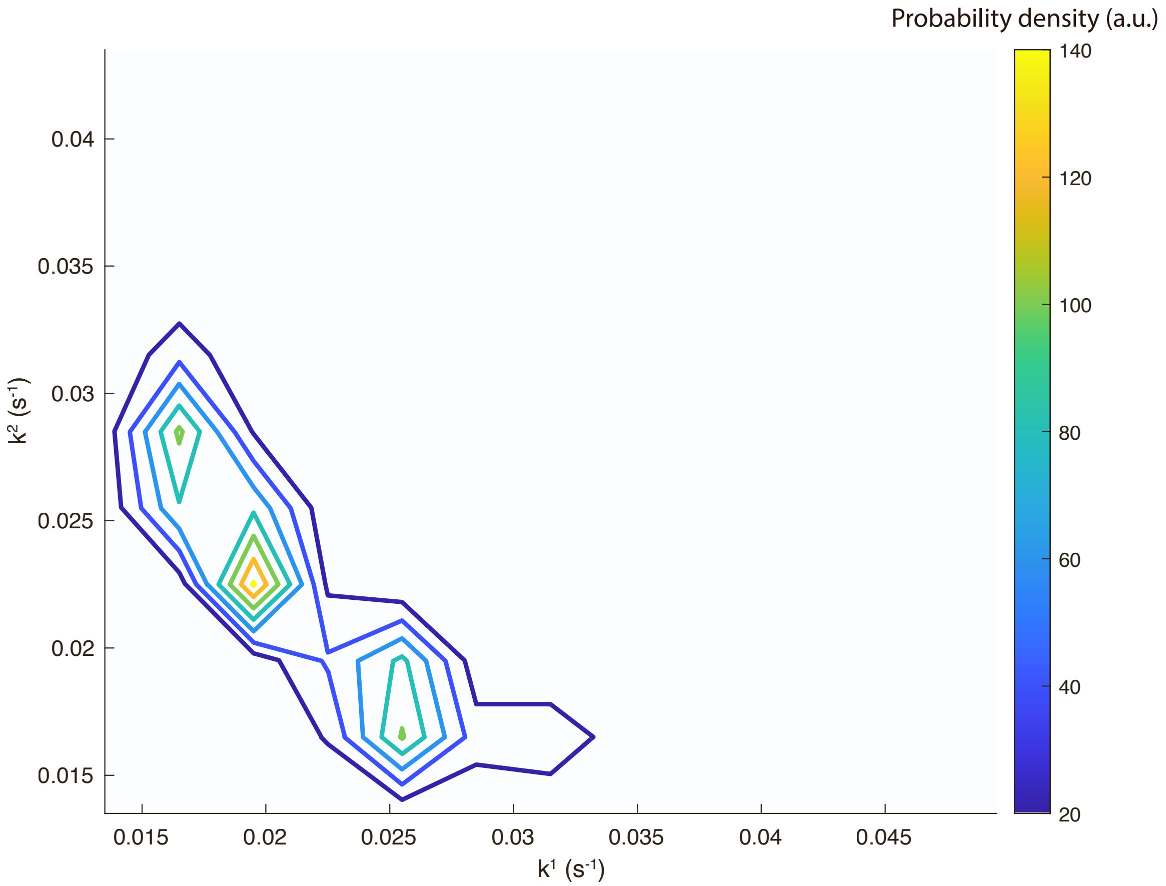

Figure S4. Joint distribution of estimated rates for influenza fusion. Lipid-mixing kinetics from influenza virus fusion were sampled using hypoexponential fits. The joint distribution of k_1 and k_2 is plotted here as a two-dimensional contour plot.

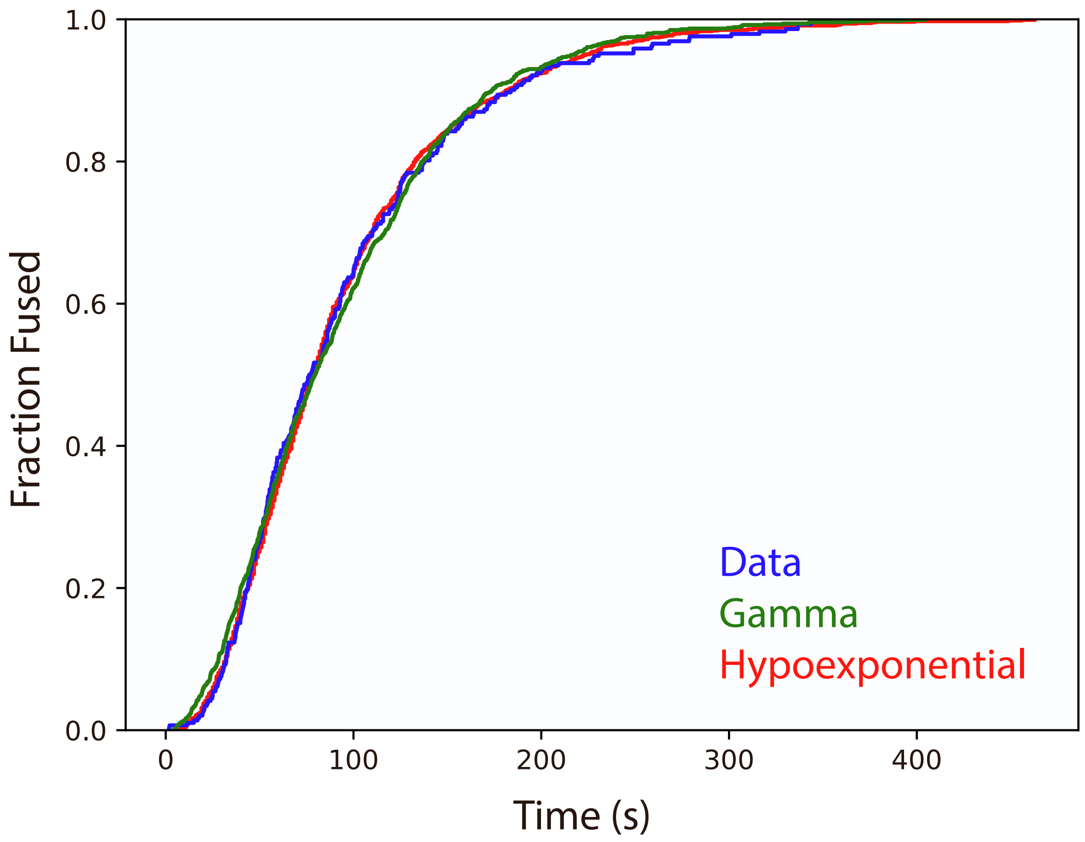

Figure S5. Comparison of gamma and hypoexponential CDFs for influenza lipid mixing kinetics. Simulated dwell times were used to reconstruct CDFs for each fitting method and plotted with the experimental data. Both methods well match the observed kinetics.

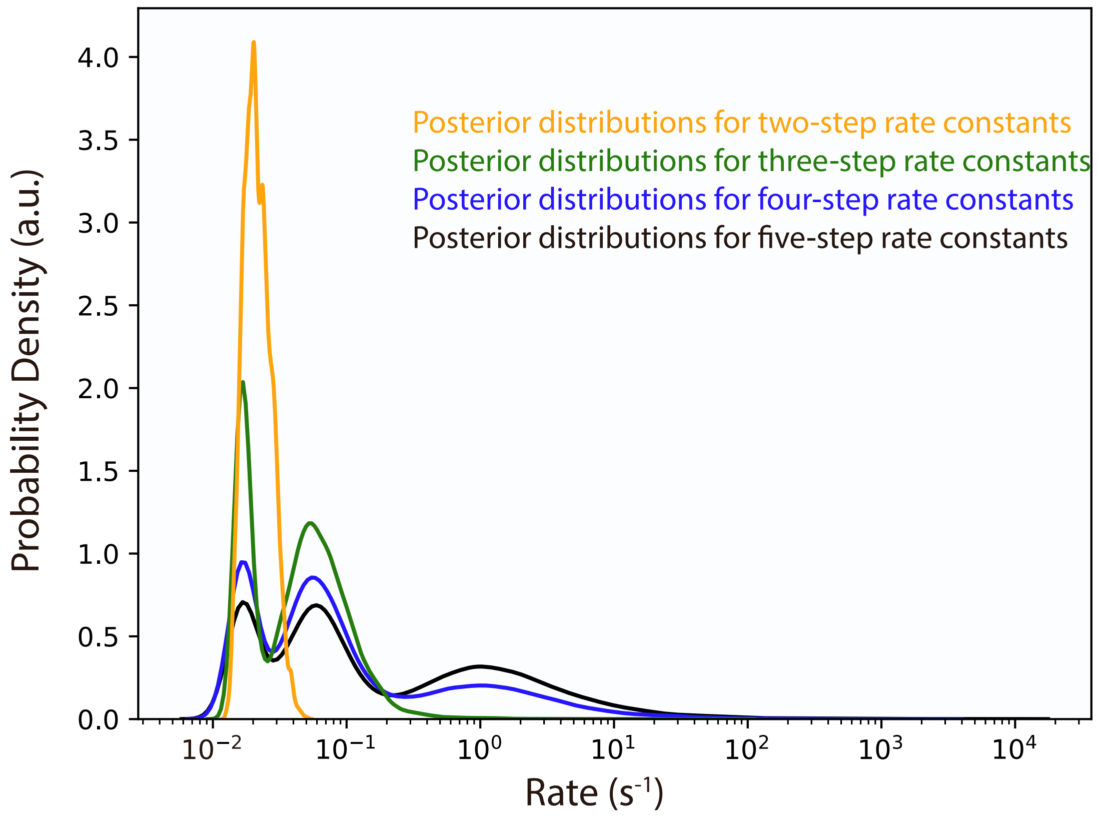

Figure S6. Posterior distributions for rate constants corresponding to 2, 3, and 4 step models for influenza fusion. Rate constants were sampled using 10,000 MCMC steps. Two-step rate constants have an essentially unimodal distribution, while three, four, and five show progressive splitting of the modes towards faster rate constants.

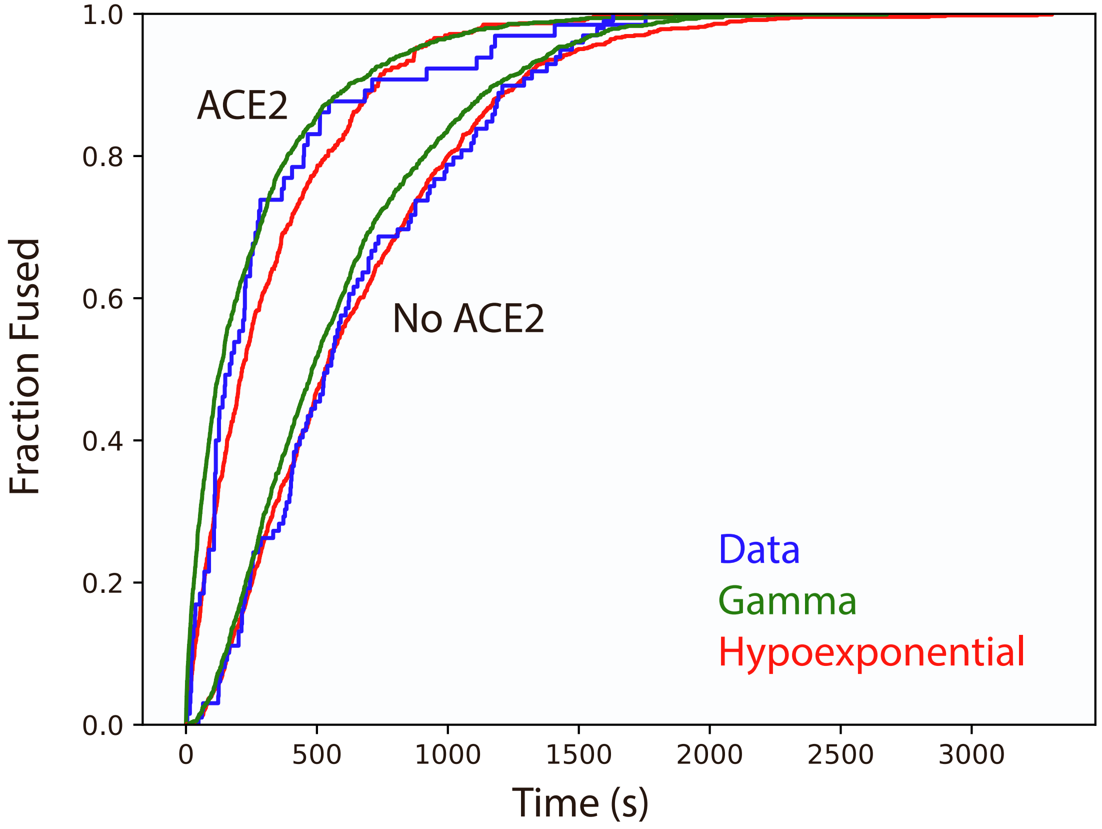

Figure S7. Comparison of gamma and hypoexponential CDFs for SARS-CoV-2 lipid mixing kinetics. Simulated dwell times were used to reconstruct CDFs for each fitting method and plotted with the experimental data. Hypoexponential distributions fit slightly better for fusion in the absence of ACE2, while gamma distributions fit slightly better for fusion in the presence of ACE2 (perhaps due to non-integer N values).

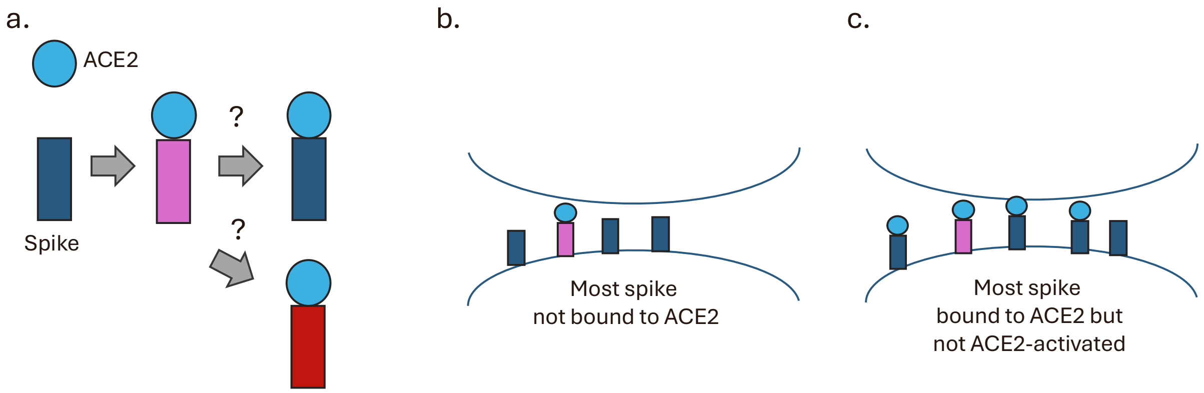

Figure S8. Schema of mixed activation scenarios. Schematized in panel (a) is the activation and inactivation of spike glycoprotein by ACE2. Activated spike is denoted in magenta. It is not known whether activated spike inactivates to baseline (teal) or to a completely unreactive spike (red). Two potential scenarios where the asymmetric model for fusion would apply are schematized in panels (b) and (c). In (b), most spike is not ACE2-bound. This is likely not true at the concentrations of soluble ACE2 used in our experiments. In (c), most spike is ACE2-bound but inactive. This could result either in mixed activation by bound, activated spike and unbound spike or, if inactive ACE2-bound spike retains baseline activity, mixed activation by bound, activated spike and bound, inactivate spike.
