# Supplementary material for: Unmasking complex kinetics in viral entry by inferring hypoexponential models
Source: Biophys J. 2025 Oct 25;124(23):4356–67. doi: 10.1016/j.bpj.2025.10.035 (PMC12709397; doi:10.1016/j.bpj.2025.10.035)
Supplement: Document S2. Article plus Supporting Material [file mmc2.pdf]

Biophysical Journal
Article

Biophysical Society

Unmasking complex kinetics in viral entry by inferring hypoexponential models

Oyinkansola Adenekan[1] and Peter M. Kasson[1,2,3,*]
[1]Department of Biomedical Engineering, University of Virginia, Charlottesville, Virginia; [2]Departments of Chemistry & Biochemistry and Biomedical Engineering, Georgia Institute of Technology, Atlanta, Georgia; and [3]Department of Cell and Molecular Biology, Uppsala University, Uppsala, Sweden

ABSTRACT Single-event completion times, such as are estimated in viral entry, offer both promise and challenge to kinetic interpretation. The promise is that they constrain underlying kinetic models much more efficiently than bulk kinetics, but the challenge is that completion times alone can incompletely determine complex reaction topologies. Gamma distributions or mechanistic models have often been used to estimate kinetic parameters for such data, but the gamma distribution relies on homogenous processes to explain the rate-limiting behavior of the system. Here, we introduce hypoexponential analysis to estimate heterogeneous kinetic processes. We demonstrate that hypoexponential models can indeed estimate rate constants separated by two to three orders of magnitude. We then apply this approach to measurements of SARS-CoV-2 entry, showing that the presence of the ACE2 receptor for SARS-CoV-2 reduces the number of rate-limiting steps but does not change the rates of these kinetic processes. We propose a kinetic model whereby SARS-CoV-2 entry is driven by a mixture of ACE2-accelerated and ACE2-independent spike protein activation events. Inferring such models requires the capability to detect heterogeneous kinetic processes, provided by robust estimation of hypoexponential distributions.

SIGNIFICANCE The biophysics of viral entry is often probed using single-event kinetics. The resulting waiting times can be analyzed to measure the rate-limiting step in entry and infer stoichiometry. Here, we introduce a family of models with improved ability to detect multiple different rate-limiting steps. We first validate the approach and then apply it to SARS-CoV-2 entry, where we motivate a new kinetic hypothesis for the role of ACE2 in viral spike activation and subsequent entry. We hypothesize that SARS-CoV-2 entry, rather than being purely ACE2 dependent when the molecule is present, is driven by a mix of ACE2-accelerated and ACE2-independent events.

INTRODUCTION

At the molecular level, biophysical processes involve stochastic transitions on a free-energy surface, which can be coarse-grained into a discrete set of states and reactions between them. Single-event experiments measure individual completion times for these reactions, reporting on mechanistic information in a way that traditional ensemble-averaged experimental techniques obscure (1). However, uncovering this mechanistic information requires more sophisticated analyses. This has been most extensively described for single-molecule experiments but also applies to a supramolecular assembly such as a virus undergoing a single set of concerted reactions. When measured end to end, single-event experiments yield a corresponding set of times from reaction initiation to reaction completion, which we refer to as dwell times (2). From a distribution of dwell times, it is possible to infer a sequence of metastable states and quantify transitions between these states (3). However, this problem is much better constrained when each metastable state is associated with a different value of an experimental observable, such as a different FRET efficiency in a single-molecule experiment. When the only experimentally accessible quantity is the end-to-end dwell time, different underlying states have the same experimental observable (4–6). Recovering the correct number of underlying states and the transitions between them thus presents an analytic challenge.

Here, we wish to recover states and rates from single-event dwell times to better understand the cell entry mechanisms of SARS-CoV-2 and influenza virus. We use single-virus fluorescence experiments to measure how

Submitted June 18, 2025, and accepted for publication October 24, 2025.
*Correspondence: kassonlab@gmail.com
Editor: Margaret Johnson.
https://doi.org/10.1016/j.bpj.2025.10.035
© 2025 The Authors. Published by Elsevier Inc. on behalf of Biophysical Society.
This is an open access article under the CC BY license (http://creativecommons.org/licenses/by/4.0/).

long it takes for SARS-CoV-2 or influenza viral envelopes to fuse with model host membranes from the time of fusion triggering. Kinetic measurements of single-viral fusion typically utilize fluorescent probes that report on viral state changes, often either lipid exchange or diffusion of a soluble probe through a fusion pore (7–10). The methods we develop are applicable to either measurement, but in this work, we use data on lipid-exchange probes and refer to lipid mixing as fusion. This sets aside for the moment the kinetics steps from lipid mixing to fusion pore opening. The kinetic parameters inferred from such measurements and analyses are then used to create mechanistic models for viral entry.

There exist several methods for quantifying kinetic states from experimental time domain data. Hidden Markov models (HMMs) (11–15) have long been applied for this purpose. Briefly, an HMM infers a discrete set of states with Markovian transitions between them, such that the time series of experimental observables is conditioned on the population of inferred states by a set of per-state emission probabilities. Both HMMs and continuous-time Markov models have been used to identify transitions between states from ion channel patch-clamp experiments (16). Similarly, HMMs have been used to identify states and lifetimes in single-molecule fluorescence resonance energy transfer (smFRET) experiments (3,17–19). HMMs are a useful tool for analyzing experimental data where each state has a distinct experimental emission pattern. Here, we address a different problem: single-event kinetics where the readout is effectively a step function $\theta(s - s_N)$, such that the start state s_0 and all intermediate states $s_{1..}s_{N-1}$ have emission 0 and the product s_N has emission 1. This is because the fluorescent reporter undergoes dequenching at the stage of lipid mixing or fusion pore opening, and the prior states $s_{0..}s_{N-1}$ have identical fluorescent yield. The solution space of HMM parameters tends to be highly degenerate for this class of problems because the intermediate states do not have distinct emissions, necessitating the development of other kinetic fitting approaches.

Previous studies fit dwell times from single-event fluorescence experimental data using a gamma function to extract reaction kinetic parameters (8,20). A gamma function describes a multistep, linear process where each step exhibits quasi-first-order kinetics with identical rates (i.e., the cumulative distribution function for an isolated step would be an exponential distribution). Researchers have used gamma analysis to develop a model of influenza entry to host cells where each kinetic step is an independent activation event between an HA spike protein on the viral surface and a target membrane mimicking the host cell (8). Since a dwell time distribution's shape is dominated by the reaction's slowest rate, gamma analysis approximates the rate constant for each step to be equal to the slowest rate. In the case where multiple distinct processes contribute to the rate-limiting behavior of a reaction, this assumption obscures some of the mechanistic complexity and thus cannot recover the maximum information from the available dwell-time distributions. Alternatively, cellular automaton models have also been used for analysis of viral entry data from single-event experiments (21–24). These models require a much more detailed starting model of the reaction, typically motivated by prior structural studies, and are thus not considered here.

In this paper, we propose recovering reaction kinetics by analyzing dwell times using the hypoexponential distribution. The hypoexponential distribution is in the same family of distributions as the gamma, but it permits a different rate for each step in a sequential process. This distribution may be well suited for estimating both number of transitions and unique rates of transition for a multistep reaction process. However, the hypoexponential distribution may have identifiability issues: as the number of states increases, it becomes harder to estimate this parameter accurately (25,26). Despite this potential issue, we hypothesize that the hypoexponential distribution permits recovery of more kinetic information from dwell time distributions than the gamma distribution alone.

Here, we first quantify the difference in performance between gamma and hypoexponential for estimating reaction kinetics, testing the limits of each as reaction rates diverge. We then apply hypoexponential analysis to single-virus fluorescence data on influenza and SARS-CoV-2 entry. We show that for two- and three-step processes, a hypoexponential distribution more accurately recovers the ground-truth reaction parameters for synthetic data. We also demonstrate that the randomness parameter, a measure developed in single-molecule enzymology (27,28), can provide highly sensitive and accurate guidance as to the number of steps in a generative kinetic scheme. We show that hypoexponential analysis recapitulates prior information on influenza membrane fusion and yields new mechanistic hypotheses for SARS-CoV-2 fusion and entry mechanisms.

Theory

We extract kinetics of biophysical reactions by fitting chemical reaction models to observed single-event reaction dwell times. One way to generate such reaction models is to decompose a complex reaction into a network where each edge represents a quasi-first-order reaction. The cumulative distribution function for each edge is thus an exponential function parameterized by a rate k_i for each edge i. Here, we consider linear processes, as shown in Eq. (1).

$$A \rightarrow X_1 \rightarrow X_2 \rightarrow X_{N-1} \rightarrow B \quad (1)$$

The biophysical rationale for this approach is that a single-barrier chemical process will display exponential kinetics of this form according to Eyring's law. A sequential process, which models a multistep biophysical reaction, thus becomes convolution of N exponential random

variables. Several distributions arise from convolution and other aggregations of two exponential random variables. Here, we focus on the gamma and hypoexponential distributions.

Previous studies fit reaction dwell times using the gamma distribution to extract kinetics. The gamma distribution is the convolution of N exponential random variables, each with an identical rate parameter $k_i = k$ for all steps i (Eq. (2)). The variables N and k are thus sufficient to parameterize the distribution. In a biophysical reaction, N corresponds to the number of free-energy barriers that contribute rate-limiting steps in the reaction. Notably, the gamma distribution assumes that k is the same for each step. Because the slowest step contributes most to the shape of the dwell time distribution, k typically reflects the slowest step in a reaction.

$$A \xrightarrow{k} X_1 \xrightarrow{k} X_2 \xrightarrow{\cdots} \xrightarrow{\cdots} X_{N-1} \xrightarrow{k} B \tag{2}$$

In this paper, we fit observed reaction dwell times using hypoexponential functions. Like the gamma function, a hypoexponential is the convolution of two or more exponential random variables. In a hypoexponential, the number of exponential random variables convolved is specified rather than inferred, and the parameters are the set of rate constants $k_{1}..k_{N}$. Eq. (3) shows a two-parameter hypoexponential parameterized by k_1, the rate of the first transition, and k_2, the rate of the second transition.

$$p(t)_{A \to B} = \begin{cases} \dfrac{k_1 k_2}{k_2 - k_1}\left(e^{-k_1 t} - e^{-k_2 t}\right), k_1 \neq k_2 \\ k_1^2 t e^{-k_1 t}, k_1 = k_2 \end{cases} \tag{3}$$

Unlike the gamma function, a hypoexponential function has an independent rate for each transition in the modeled process. Because the hypoexponential specifies the number of transitions, we can estimate hypoexponential distributions with increasing numbers of transitions, $\tilde{N} = 1,2,\ldots$ and select N by identifying the model with the lowest corrected Akaike information criterion (AICc) (29,30) or via the randomness parameter (27,28,31) as described below. We estimate the rate probabilities using Metropolis-Hastings Markov chain Monte Carlo (MCMC) sampling.

MATERIALS AND METHODS

Single-virus fluorescence experiments

Single-virus kinetics of influenza membrane fusion were performed according to previously published protocols (32) with the exception that the microfluidic flow cell was maintained at 32°C. X-31 (A/Aichi/1968 H3N2) influenza virus was bound to GD1a glycosphingolipid receptors displayed on liposomes immobilized in a microfluidic flow cell, and fusion was triggered by buffer exchange to pH 5.0. Fusion was assessed by dequenching of Texas Red-DHPC dye loaded into the viral envelope at a quenching concentration. Single-event dwell times—the intervals measured between pH drop to initiate fusion and fluorescence dequenching—were provided by Robert Rawle.

Single-virus kinetics of SARS-CoV-2 virus-like-particle fusion were previously reported (33), and the data are reanalyzed here. Kinetics were measured using fluorescently labeled pseudovirus fusing to liposomes, with attachment mediated by synthetic DNA-lipid attachment factors and fusion triggered using soluble protease. Experiments were performed with and without soluble ACE2 receptor. These data take the form of single-virus dwell times: the interval between fusion triggering of bound virus using a soluble protease and the fluorescence dequenching that serves as a real-time fusion signal.

Data simulation and modeling

For initial validation, we generated synthetic data corresponding to known ground-truth values of N and k_i using the Gillespie algorithm (34,35) implemented in the *pysb* and *bionetgen* Python packages and APIs. We simulated 300 dwell times for each set of ground truth values to emulate a typical experimental data set. Plotted data were generated using a 1-s discrete time step (time resolution), matching the experimental acquisition interval for the SARS-CoV-2 data. We also performed sensitivity analyses with time steps from 1 s to 0.01 s.

Fitting of hypoexponential distributions to both synthetic and experimental data was performed using MCMC implemented with a simple Metropolis-Hastings algorithm written in Python. The algorithm proposes a set of values for the rate parameters and then accepts or rejects the proposal based on its likelihood given the observed data. Code can be found on GitHub: https://github.com/kassonlab/hypoexponential-analysis. Gamma distributions were fit to the data using either MCMC sampling or a maximum-likelihood approach implemented in previously published MATLAB code (36). For MCMC, convergence analyses were performed using 1 to 100 independent sampling runs, each with 1000 to 50,000 iterations. It was found that a single run of 50,000 iterations was amply sufficient for convergence in reproducing synthetic data, and these settings were used for evaluation of experimental data. The first 100 (for 1000-step runs) to 1000 iterations (for ≥10,000-step runs) were labeled as burn-in, and the remainder were taken as the estimate of the posterior rate distribution. Estimated kinetics were also compared with observed kinetics by sampling dwell times from the estimated model, compiling them into an empirical CDF (eCDF), and comparing this simulated eCDF to one generated from the experimental observations (or synthetic data in model validation tests).

Estimating the number of steps *N*

The number of steps was estimated either by directly comparing the models using the corrected AICCc or the model-free randomness parameter. AICc and related information criteria (29,30,37) all balance the likelihood of a model against its complexity. We therefore performed hypoexponential sampling with $N = 1,2,3,4,5$. AICc model selection then selects the model with the lowest AICc value. A pilot test was performed using Bayesian information criterion model selection, which performed similar but slightly worse than AICc in this case, and the two model selection criteria are further compared in Fig. S1. The randomness parameter is derived from the relative variance of single-event waiting times and is defined as follows: $r = (\langle t^2\rangle - \langle t\rangle^2)/\langle t\rangle^2$, where t represent the single-event dwell times (28,31). $N_{\min} = 1/r$ is the minimum number of kinetic steps in a linear reaction scheme required to reproduce the observed waiting time distribution.

Evaluating hypoexponential and gamma fits

To facilitate parallel evaluation of gamma fits and MCMC estimation of hypoexponential distribution parameters, we sampled waiting times from each estimated model and replotted CDFs from these estimates to compare to

observed (experimental or synthetic) data. These constitute the curves shown in the CDF figures. Although the full posterior distribution is most informative, we extract maximum-likelihood estimates as the sampled rate parameter tuples corresponding to the highest posterior likelihood. Confidence intervals are also calculated from the posterior distributions and presented for rate estimates on experimental data.

We evaluated the performance of both hypoexponential and gamma fits (k and N) on synthetic data using percent error: $\mathrm{error_k} = \mathrm{abs}(k_{\mathrm{ground\ truth}} - k_{\mathrm{estimated}})/k_{\mathrm{ground\ truth}} \times 100$, and absolute error: $\mathrm{error_N} = \mathrm{abs}(N_{\mathrm{ground\ truth}} - N_{\mathrm{estimated}})/N_{\mathrm{ground\ truth}}$. Hypoexponential models return an integer number of steps, whereas gamma fits return a real number. We use the error of the mode for hypoexponential models because a fractional number of steps is not meaningful for a hypoexponential distribution, whereas it is for the gamma distribution.

RESULTS AND DISCUSSION

Extracting kinetic parameters on viral entry process by fitting data from single-virus fluorescence experiments to reaction models

We briefly describe the experimental design and analytical goals, followed by validation on synthetic data and application to problems in viral entry. We use single-virus optical microscopy where fusion kinetics are measured using a fluorescent reporter of lipid mixing. The unfused virus and all intermediates until the process of interest occurs have baseline fluorescence, and the fused conjugate has elevated fluorescence. The approach is schematized in Fig. 1, and details on the experimental protocol have been given in a recent methods paper (10). Briefly, target liposomes are immobilized in a flow cell, and viral particles are added and bound to them. Binding occurs either via endogenous receptors or synthetic DNA tethers (36). Each viral particle that fuses generates an abrupt increase in fluorescence. The interval from triggering to fusion is then extracted as the waiting time to fusion. Collating these waiting times from many individual particles yields a distribution of dwell times that can be represented using an eCDF. We model this curve as a biophysical reaction that starts in an unfused state and traverses through a number of intermediate states N before reaching the final fused state. We fit the aforementioned reaction model to dwell times from single-virus fluorescence experiments to estimate N and rate constants k_i for transitions between states.

Hypoexponential sampling outperforms gamma analysis in reconstructing generative reaction models for synthetic data

In evaluating the utility of hypoexponential sampling, we first asked whether it could outperform gamma analysis when the ground truth reaction model was known. To that end, we created synthetic data using reaction models consisting of two or three kinetic steps and where the k_i values varied from identical to 100,000-fold different. Rate values were chosen to cover the ranges anticipated for influenza and SARS-CoV-2 single-virus fusion kinetics (8,21,33,36,38). Synthetic data were created by sampling dwell times using the Gillespie algorithm (34). We then compared the results of hypoexponential sampling and gamma analysis on these synthetic data. Fig. 2 shows eCDFs for three synthetic data examples and the corresponding gamma and hypoexponential fits. In general, both gamma and hypoexponential distributions produced near-equivalent fits when the ground truth rate constants were identical or when they were so different that the fast step is no longer rate limiting. Both overall fit the CDFs through the entire range, but subtle differences were appreciable when the fast and slow rates differed by a moderate amount. Our goal here is not gross reproduction of the CDF, but rather accurate recovery of mechanistic information. In subsequent figures, we compare gamma and hypoexponential performance on individual rate estimates.

Performance on two-step reactions

As shown in Fig. 3, hypoexponential sampling better recovers both rates and number of steps in the ground truth reaction model than does gamma analysis. Interestingly, for the slower step, hypoexponential and gamma analysis recover rates with similar accuracy. Hypoexponential sampling recovers about 90% of rates with less than 20% error, whereas gamma fitting recovers about 70% of the slower rates at the same error level. For the faster step,

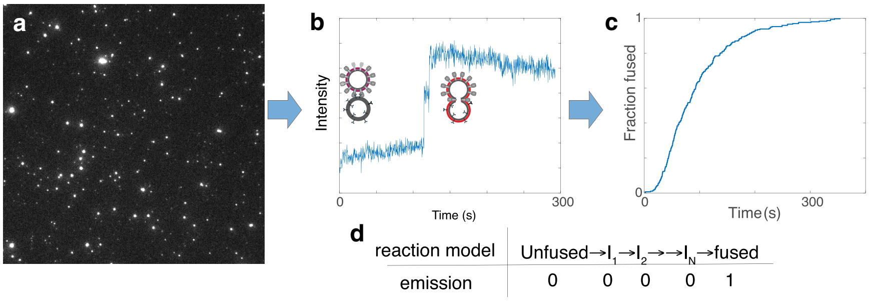

FIGURE 1 Representative data showing (*a*) a fluorescence micrograph of influenza virus undergoing fusion, (*b*) a single-virus intensity-time trace corresponding to one spot in the image with unfused and fused membranes schematized (*b*), (*c*) the CDF compiled from these trace, and (*d*) a linear reaction model with corresponding 0 and 1 emissions denoting pre- and postjump intensity traces.

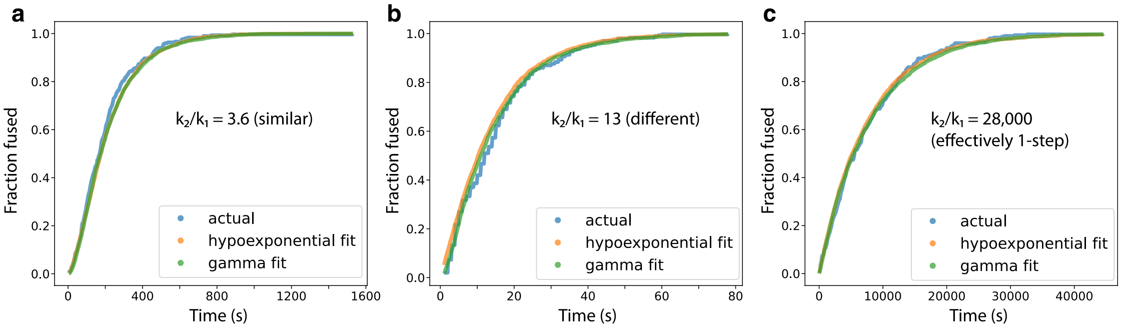

FIGURE 2 Cumulative distribution functions and fits plotted for three scenarios, where k_1 and k_2 are similar (*a*), where they differ substantially (*b*), and where they differ sufficiently that k_2 is no longer rate limiting (c). Rate constants used were 5.92e−3 and 2.13e−2 s^{-1} (*a*), 7.65e−2 and 0.988 s^{-1} (*b*), and 1.28e−4 and 3.55 s^{-1} (*c*). Hypoexponential estimates are from 1000 MCMC samples.

however, hypoexponential analysis substantially outperformed gamma analysis. Although both methods display increased error as rates deviate from one another, hypoexponential models are less affected by this. For the hypoexponential models, percent error is consistently above 50% when rates are more than 100-fold apart. For gamma recovery of the faster step, percent error is always above 50% when rates are not identical. This highlights the weakness of gamma fitting: because its underlying model assumes identical rates, it is fundamentally unable to recover rate constants that differ substantially from each other.

Recovery of the number of steps (N) displays a similar trend: worse recovery as the rates deviate more but better performance with hypoexponential sampling than gamma. Hypoexponential sampling generally recovers N accurately for rates differing less than 1000-fold, whereas gamma fitting only recovers N when rates are within 100-fold. Interestingly, hypoexponential models poorly recover N for pairs with similar rates >0.1 s^{-1} when the sampling rate was 1 s^{-1}. Overall, hypoexponential sampling outperforms gamma fitting in recovering rates and number of kinetic steps (55% vs. 33%) in two-step synthetic reaction schemes. These synthetic experiments establish hypoexponential sampling as a useful tool for extracting kinetics, particularly when rates differ within 100- to 1000-fold. Poorer recovery with very fast rate constants likely stems from the discrete sampling frequency—a faster sampling rate, corresponding to a faster experimental acquisition rate, yielded better recovery in sensitivity analyses of such scenarios. The

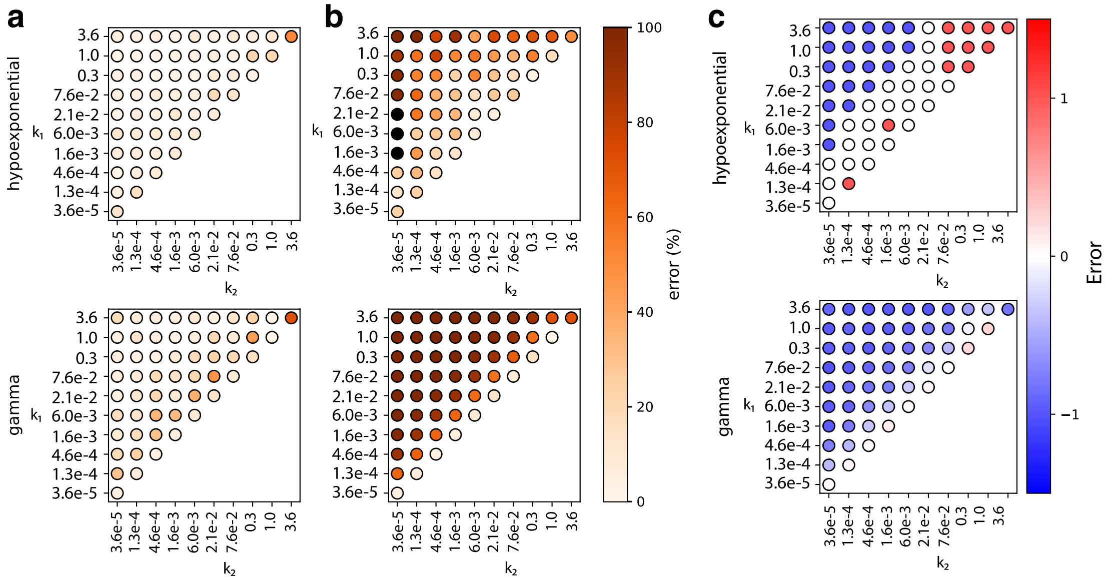

FIGURE 3 Performance comparison of gamma and hypoexponential fits for two-step reactions. Three independent sets of synthetic data for each pair k_1 and k_2 values listed were fit using either gamma or hypoexponential MCMC approaches. Mean percent error in estimated k_1 (slow rate) values (*a*), k_2 (fast rate) values (*b*), and absolute error in estimated number of steps N (*c*) are plotted. Black circles denote error of >100%. Hypoexponential fits outperform gamma in all three recovery tasks except when the two rates are equal. Data are replotted as a function of k_2/k_1 ratio in Fig. S2. Hypoexponential estimates are from 1000 MCMC samples.

dependence of error on sampling rate is shown in Fig. S3. The sampling rates used experimentally, 3.47 s^{-1} for influenza and 1 s^{-1} for SARS-CoV-2, are likely sufficient to determine the rate constants as estimated below. Faster kinetic processes would demand faster sampling rates. The reason not to image arbitrarily fast is that excessive illumination of the fluorescent reporters used can perturb fusion kinetics (32).

Performance on three-step reactions

We also tested hypoexponential sampling versus gamma analysis on three-step synthetic reaction schemes (Fig. 4), and it again displayed better recovery of kinetic parameters. For the slowest step, hypoexponential sampling slightly outperforms gamma analysis: recall of 89% to $\leq$20% error versus 63% to $\leq$20% error. Similar to the two-step analysis, as the rates deviate from one another, hypoexponential sampling recovers the middle rate much better than gamma: 58% to within 50% error, versus 25% to within 50% error. For the third and fastest step, hypoexponential sampling recovers 30% to $\leq$50% error, whereas gamma fails in all cases when the rates are nonidentical. These results accentuate the trends from the two-step data: both approaches are challenged by multistep reactions with highly differing rates, but hypoexponential sampling performs better in these scenarios. This is in line with the gamma function's underlying model of identical rates: it consistently recalls the slowest step in a reaction but cannot estimate differing rates. Finally, hypoexponential sampling generally recovers N accurately when rates are within 100-fold of one another, whereas gamma only recovers N accurately when rates are within 10-fold, showing the same trend as the two-step reactions but increased stringency due to the added kinetic complexity. Although hypoexponential sampling recovers kinetics more accurately than gamma for three-step kinetic systems, the reliability in recovering the fastest rate is low. This may reflect a statistical limit in reconstructing diverse rates and a requirement for substantially greater data volumes in these cases.

For two-step kinetic systems, the highest accuracy in recovering number of steps is achieved using the randomness parameter. The randomness parameter is thus directly calculated from single-event observations and is not dependent on an analytic model. $N_{min} = 1/r$ reports on the minimum number of steps in a linear scheme required to generate the observed waiting times (2,27). Not surprisingly, ceil(N_{min}), the smallest number of integer steps satisfying N_{min}, performed better than either AICc model selection on hypoexponential sampling or the N parameter from gamma fitting on our synthetic data test set (Fig. 5). Interestingly, the failures for this estimator occurred on a number of equal or near-equal rates, suggesting that an optimal estimator might account for some stochastic noise and take the form ceil($N_{min} - \epsilon$) for some tolerance hyperparameter ϵ. The rationale for this formulation is that if stochastic error is excluded, a N_{min} of 2.2 would require at least three steps, so ceil(N_{min}) encodes this dependence. The ϵ is introduced to account for stochastic error, in effect differentiating N_{min} of 2.0 $\pm$ ϵ from N_{min} of 2.2.

Estimating multistep kinetics of influenza viral entry

Influenza virus undergoes a multistep reaction to fuse its envelope with endosomal membranes in the cell and release the viral genome into the cytoplasm. This fusion

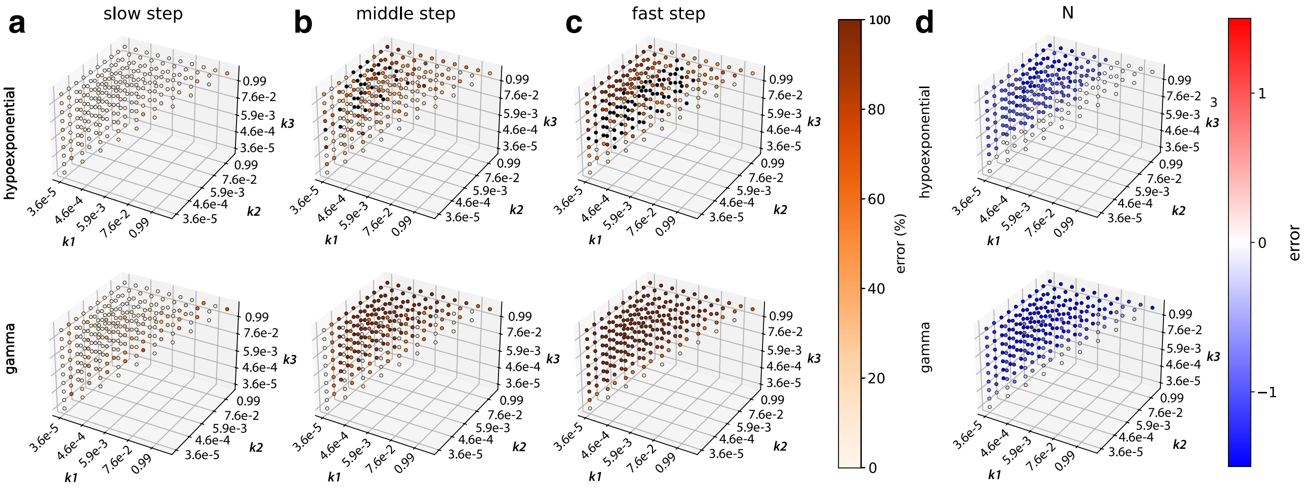

FIGURE 4 Performance of gamma and hypoexponential fits on synthetic data from three-step reactions. Percent error in recovery of the slowest, middle, and fastest rate constants is plotted in (*a*)–(*c*); absolute error in recovery of the number of steps is plotted in (*d*). Black circles denote an error >100%. Hypoexponential fits outperform gamma fits in cases where the rates are moderately different, substantially extending the range of accurate rate recovery. Hypoexponential estimates are from 1000 MCMC samples.

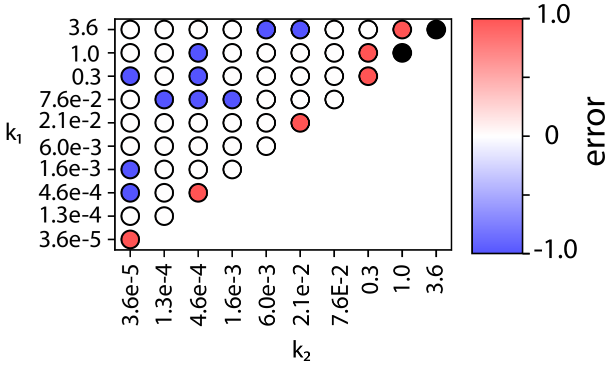

FIGURE 5 The randomness parameter provides a robust estimate of the number of steps in a heterogeneous kinetic process. Shown is the median error in the number of steps using ceil(N_{min}) for two-step processes with varying rates, computed over three independent synthetic data sets. As can be seen, this estimator overall outperforms either AICc on hypoexponential sampling or gamma fitting but has some increased error along the "identical rate" diagonal. Black points denote an error >1.0, which in this case is produced by rates too fast for the 1-s sampling interval.

reaction has been extensively studied using both bulk and single-virus kinetics experiments (7,8,21,36,39). Prior fitting of influenza fusion kinetics at the single-virus level has primarily utilized either gamma functions or more mechanistically detailed cellular automaton models (8,21,22,40). The randomness parameter has also been used as a means to determine the minimum number of sequential steps in the underlying kinetic scheme (41,42). Lipid mixing, or exchange of labeled lipids between the influenza virion and a target membrane, was modeled as two to four identical steps, with the greatest likelihood being three identical steps in most cases (8). Based on mutational experiments, the rate-limiting step was linked to pH-dependent release of the hemagglutinin fusion peptide (21).

We applied hypoexponential sampling to better understand the kinetic steps leading to influenza entry. The AICc-selected hypoexponential model for influenza lipid mixing using the data in Fig. 6 involved two kinetic steps, which agrees well with gamma fits and randomness parameter analysis on this data set.

For a more detailed comparison, we leveraged the MCMC sampling process to examine posterior probability distributions and confidence intervals for rates rather than simple maximum likelihood estimates. Those are plotted in Fig. 6 *b* to correspond to the maximum-likelihood estimates in Fig. 6 *a*. For lipid-mixing data, posterior distributions corresponding to the number of kinetic steps predicted by the randomness parameter show N indistinguishable rate constants $k_i = k_j = k_N$. Models corresponding to >N kinetic steps show N rate constants slightly slower than k_N and the remaining rate constants much faster than k_N (Fig. S6). These results, as well as the synthetic data, demonstrate that hypoexponential sampling well captures two-step and typically three-step processes and that its failure modes involve overreporting of differing rate constants $k_i \neq k_j$ rather than overreporting of identical rate constants $k_i = k_j$.

Hypoexponential analysis suggests an "asymmetric" model for ACE2 action in SARS-CoV-2 fusion

We next applied hypoexponential sampling to help understand the rate-limiting steps in SARS-CoV-2 fusion kinetics. Briefly, SARS-CoV-2 entry involves spike protein binding

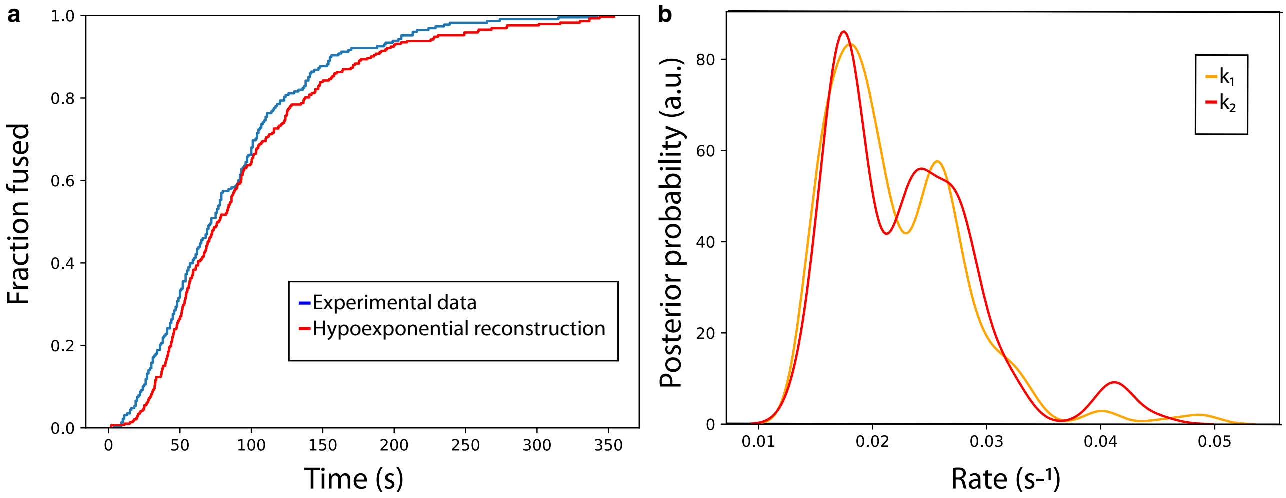

FIGURE 6 Hypoexponential fit to influenza lipid mixing data. Plotted in (*a*) are empirical CDFs for the observed lipid-mixing times (*blue*, 292 data points) and the hypoexponential fit (*red*). Plotted in (*b*) are posterior probability density estimates for k_1 and k_2 in the hypoexponential fit. Maximum-likelihood rate estimates are 0.019 s^{-1} (95% CI 0.15–0.34) and 0.023 s^{-1} (95% CI 0.14–0.41). The joint distribution of the rate probabilities is plotted in Fig. S4, and hypoexponential and gamma CDFs are compared in Fig. S5. Hypoexponential estimates are from 50,000 MCMC samples.

to cellular ACE2 receptors (43,44) and proteolytic activation of the spike to trigger fusion (45,46). ACE2 facilitates a conformational change in the spike protein (47) that is linked to fusion. Surprisingly, however, single-virus fusion experiments found that ACE2 is not strictly required for SARS-CoV-2 fusion (33). In this case, DNA-lipid tethers (48) were used to attach SARS-CoV-2 virus-like particles in the absence of ACE2 receptor, and fusion kinetics after proteolytic triggering were measured with and without the addition of soluble ACE2. In the initial report, the resulting data were analyzed using gamma distributions (33). This analysis yielded 1) N$\sim$2 or two rate-limiting steps in the absence of ACE2 and 2) N$\sim$1 or one rate-limiting step in the presence of ACE2, but they did not further illuminate the chemical nature of this difference. Here, we fit hypoexponential functions to the previously measured single-virus fusion kinetics to generate testable mechanistic hypotheses regarding the rate-limiting steps in SARS-CoV-2 entry.

Hypoexponential analysis agrees with the fundamental finding from gamma analysis and provides additional information on SARS-CoV-2's mechanism of entry. Both approaches predict the most likely model to be one kinetic step with ACE2 and two kinetic steps without. Interestingly, the rates predicted from hypoexponential sampling where k_1 and k_2 can vary freely were 0.0031 and 0.0033 s^{-1} without ACE2 and 0.00338 s^{-1} with ACE2 (Fig. 7). These are highly similar both to each other and to the rates predicted from gamma fitting where k_1 and k_2 are constrained to be equal: 0.0030 s^{-1} either with or without ACE2.

Perhaps most extensively explored for influenza, viral fusion proteins are believed to have an additive effect in driving membrane fusion: proteins activate for fusion independently with identical rate constants, and fusion requires the activity of multiple activated viral glycoproteins. This interpretation was first advanced based on kinetic modeling and careful mutagenesis in bulk experiments (49–51). More recently, it has been more directly supported by initial single-virus fusion kinetics (8), measurements of the effects of neutralizing antibodies (52), and kinetic fitting of cellular automata models that encode these structural assumptions directly (21,22). For influenza, we have observed that the apparent number of rate-limiting steps can vary with temperature (42), which led us to propose a variable-stoichiometry model for influenza fusion. In this model, there is some underlying free energy barrier for lipid-mixing in fusion $\Delta G_F^{\ddagger}$. Each viral glycoprotein activation event creates an intermediate ΔG_S beyond the starting state, such that more independent activation events reduce the remaining free-energy barrier for fusion. This model was created for influenza but should in theory apply for any viral fusion event where glycoproteins undergo independent activation, fusion likely involves more than one activated glycoprotein, and the activated glycoproteins form a loose rather than crystallographically ordered complex to drive lipid mixing and subsequent fusion.

Based on this, we propose the following kinetic model for SARS-CoV-2 fusion kinetics (Fig. 8). Similar to models for influenza discussed in our prior work (42), we formulate a variable-stoichiometry model for fusion protein activation, where docked, unfused virus has an activation free energy for fusion of $\Delta G_F^{\ddagger}$. We note that in this case, we use a fluorescent reporter for lipid mixing, so all events subsequent to lipid mixing are lumped into the "F" or fused state, and kinetic steps relating to fusion pore opening are not explicitly examined. This can be done using simultaneous imaging of lipid-mixing and content-exchange reporter dyes (8,53) but was not available for this data set. Each viral protein activation event occurs with a rate k_S and lowers the activation barrier to fusion by ΔG_S, with the corresponding k_{Fi} rate constants as schematized in the figure. The relative probability of outbound transitions (and thus relative flux) from any given intermediate is thus given by the ratio of first-order rate constants. Hypoexponential sampling can reliably recover rates that differ by less than $\sim$100$\times$ in a three-step process. Therefore, estimation of SARS-CoV-2 fusion as a two-step process in the absence of ACE2 means that either 1) the maximum-flux pathway from U to F in this model is U$\rightarrow$US$_1\rightarrow$F or 2) that it is U$\rightarrow$US$_1\rightarrow$US$_2\rightarrow$F and $k_{F2}/k_S \geq 100$. Since we estimate the two steps to have near-identical rates in the absence of ACE2, U$\rightarrow$US$_1\rightarrow$F is unlikely because $k_S \sim k_F$ would result in an approximately 1:1 mixture of flux through the U$\rightarrow$US$_1\rightarrow$F pathway and the

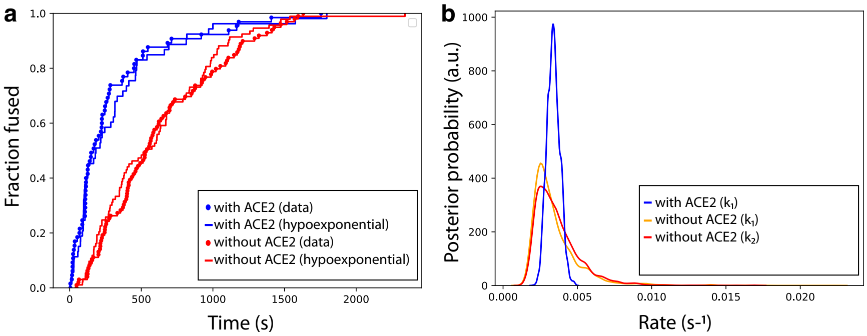

FIGURE 7 Kinetics of SARS-CoV-2 entry with and without ACE2. Empirical CDFs and hypoexponential fits are plotted in (*a*) for 65 experimental observations with ACE2 and 99 without, whereas posterior probability density estimates for the rates are plotted in (*b*). Maximum-likelihood hypoexponential parameter fits are with ACE2: $k = 0.00338$ s^{-1} (95% CI 0.0026 to 0.0042), $N = 1$ and without ACE2: $k_1 = 0.00310$ s^{-1} (95% CI 0.0019 to 0.0068), $k_2 = 0.00327$ s^{-1} (95% CI 0.0020 to 0.0072), $N = 2$. Hypoexponential and gamma CDFs are compared in Fig. S7. Hypoexponential estimates are from 50,000 MCMC samples.

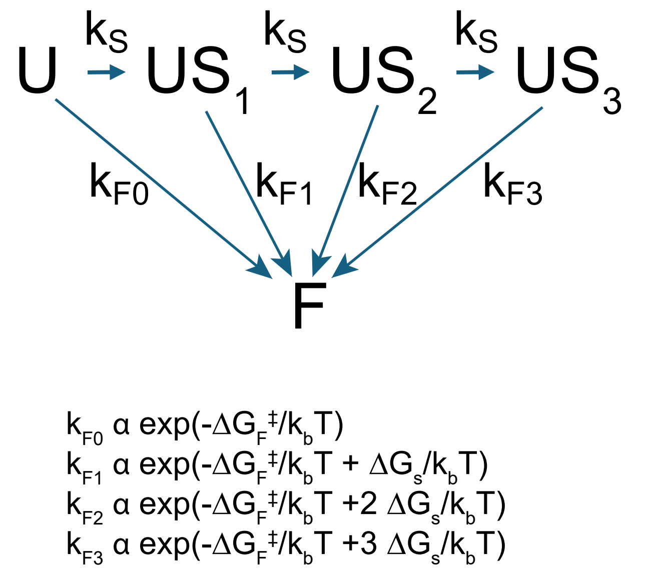

FIGURE 8 Kinetic scheme for fusion. U, US_n, and F represent states in the scheme. $\Delta G_F^{\ddagger}$ is the activation energy for fusion, ΔG_s is the free energy contributed by each activated spike toward fusion, k_b is Boltzmann's constant, and T is temperature. In this case, fusion is measured by a lipid-mixing probe only, so kinetic events from lipid mixing to full fusion pore are lumped into the "F" state and not differentiated explicitly.

$U \rightarrow US_1 \rightarrow US_2 \rightarrow F$ pathway. We therefore conclude the most likely pathway is $U \rightarrow US_1 \rightarrow US_2 \rightarrow F$ with $k_{F2}/k_S \geq 100$. This implies that $\Delta G_F^{\ddagger} = 2\Delta G_S + \boldsymbol{\delta}$, where $\boldsymbol{\delta} < \Delta G_S$, so that $k_{F2} >> k_S$ and $k_{F1} < k_S$. This would also be consistent with ΔG_A, the spike activation free energy corresponding to k_s, $< \Delta G_S$, as might be expected for a kinetically spring-loaded fusion protein (54).

We then consider the kinetic data in the presence of ACE2. Structural and smFRET data suggest that ACE2 affects the activation of SARS-CoV-2 spikes but would be unlikely to alter ΔG_F (45,47,55,56), the energetic contribution of these spikes to fusion. Therefore, our favored hypothesis is that ACE2 affects k_S nonuniformly: the most probable pathway in our scenario is that one spike activation event is ACE2 accelerated and not detected as rate limiting, whereas a second spike activation event is "not" ACE2 accelerated and remains at the original rate k_S. The data do not exclude that these two events could represent different molecular processes that have indistinguishable rates. However, given the available information, the most parsimonious explanation is that two processes with indistinguishable rates represent similar molecular processes—here postulated to be spike activation events, particularly since additional data support such an interpretation for other viruses such as influenza (52).

This seemingly counterintuitive "asymmetric" scenario could arise from two key properties of ACE2-spike interactions: it may be that the number of ACE2s in the viral contact region is typically low, and thus the encounter between a second spike and ACE2 may be slow compared with spontaneous activation of that spike. Alternatively, ACE2 has been observed to drive spontaneous inactivation of spike trimers that do not immediately proceed to fusion (33). This inactivation process would limit the time window for recruitment of a second spike, making it unlikely that two spikes remain active during the same time window. Experimentally, adding a higher concentration of soluble ACE2 returned fusion kinetics to match those without ACE2 (33), which would favor a model where inactivation of spike-bound ACE2 is the controlling factor for soluble-ACE2 experiments rather than sparse ACE2 at the interface. Dissociation of ACE2 from spike is at approximately 3×10^{-3} s^{-1} (57), so both fusion and inactivation timescales are likely faster than dissociation (33). These models are schematized in Fig. S8. Which of these two is physiologically relevant remains to be determined. We propose future experiments to resolve this by measuring SARS-CoV-2 fusion kinetics as a function of ACE2 membrane density. Such experiments, in combination with experimentally derived estimates of ACE2 inactivation timescales, should further constrain mechanistic models.

CONCLUSION

In any biomolecular process, the rate-limiting steps could be singular, multiple and identical, or multiple and heterogeneous. When available data consist of single-event dwell times (reporting only on reaction completion), preexisting approaches often differentiate poorly between the last two cases. We have shown that hypoexponential fits can reliably extract heterogeneous rate constants over a two- to three-order of magnitude range. Since this corresponds to approximately 7–10 k_bT, this permits a reasonable capability to probe different mechanistic steps. Even if inclusion of heterogeneous rates at the high end of this detection range contributes only slightly to recovering the measured dwell-time distribution, their detection can have important mechanistic consequences.

Hypoexponential distributions do have some limitations. They are not well suited to directly modeling reactive flux through multiple pathways, for instance substantial occupancy of different pathways in Fig. 8 under a single set of experimental conditions. Prior work on the randomness parameter has shown that estimating linear reactions of four to eight steps can become highly susceptible to noise (41), although it is possible that large sample sizes can overcome this limitation. Similarly, prior theoretical work suggests that completion times for hypoexponential and similar distributions are well approximated by either an exponential distribution or a delta function at large numbers of steps (58). Thus for substantially more complex pathways, additional experimental observables and potentially additional analysis methods become necessary to determine

mechanistic details. For the range of pathways we consider here, hypoexponential sampling has the ability to provide substantial new insight.

In this work, we demonstrate a case where definitively ruling out heterogeneous rates within this range yields biological insight. Prior analysis of the role of ACE2 in promoting SARS-CoV-2 fusion had identified a change in the number of rate-limiting steps but could not differentiate whether the component steps were all identical or involved different mechanistic processes. The ability to detect heterogeneous rates, and in this case, the uniformity of rates detected, helps us assign all the identical rate-limiting steps to ACE2-mediated SARS-CoV-2 spike activation. This yields a novel and experimentally testable hypothesis: that SARS-CoV-2 fusion involves one ACE2-accelerated activation event and one ACE2-independent activation event. In the absence of ACE2, both activation events are kinetically and, we propose, mechanistically identical to the ACE2-independent activation event. This seemingly counterintuitive prediction could result if the likelihood of two spikes being simultaneously activated by ACE2 molecules was small and if the rate of encounter between a second spike and a second ACE2 is slower than the rate of ACE2-independent activation (estimated here at 0.003 s^{-1}). Such an event could also occur if one spike undergoes spontaneous activation before ACE2 encounter by the second spike. This can be tested and compared with physiological virus-cell encounters by experimentally tuning the virus-ACE2 encounter rate.

DATA AND CODE AVAILABILITY

Source code is available at https://github.com/kassonlab/hypoexponential-analysis. SARS-CoV-2 fusion data are available from the Zenodo repository associated with the original publication: https://doi.org/10.5281/zenodo.7853006. Influenza single-event dwell-time data are available as part of the GitHub source repository.

ACKNOWLEDGMENTS

The authors thank Robert Rawle for helpful discussions and influenza single-event dwell-time data. Marcos Cervantes provided single-event dwell-time data for SARS-CoV-2 fusion. This work was supported by NIGMS R01GM138444 to P.M.K. Work in Sweden was supported by the Knut and Alice Wallenberg Foundation KAW 2020.0209. O.A. was also supported by a traineeship under NSF 2021791.

AUTHOR CONTRIBUTIONS

O.A. and P.M.K. designed the project, developed methodology, analyzed data, and wrote the paper.

DECLARATION OF INTERESTS

The authors declare no competing interests.

SUPPORTING MATERIAL

Supporting Material can be found online at https://doi.org/10.1016/j.bpj.2025.10.035.

REFERENCES

1. English, B. P., W. Min, …, X. S. Xie. 2006. Ever-fluctuating single enzyme molecules: Michaelis-Menten equation revisited. *Nat. Chem. Biol.* 2:87–94. https://doi.org/10.1038/nchembio759.
2. Kou, S. C., B. J. Cherayil, …, X. S. Xie. 2005. Single-molecule Michaelis-Menten equations. *J. Phys. Chem. B.* 109:19068–19081. https://doi.org/10.1021/jp051490q.
3. van de Meent, J. W., J. E. Bronson, …, R. L. Gonzalez, Jr. 2014. Empirical Bayes methods enable advanced population-level analyses of single-molecule FRET experiments. *Biophys. J.* 106:1327–1337. https://doi.org/10.1016/j.bpj.2013.12.055.
4. Liu, S., G. Bokinsky, …, X. Zhuang. 2007. Dissecting the multistep reaction pathway of an RNA enzyme by single-molecule kinetic "fingerprinting". *Proc. Natl. Acad. Sci. USA.* 104:12634–12639. https://doi.org/10.1073/pnas.0610597104.
5. Huang, W. Y. C., S. Alvarez, …, J. T. Groves. 2021. Relating cellular signaling timescales to single-molecule kinetics: A first-passage time analysis of Ras activation by SOS. *Proc. Natl. Acad. Sci. USA.* 118:e2103598118. https://doi.org/10.1073/pnas.2103598118.
6. Santoso, Y., J. P. Torella, and A. N. Kapanidis. 2010. Characterizing single-molecule FRET dynamics with probability distribution analysis. *ChemPhysChem.* 11:2209–2219. https://doi.org/10.1002/cphc.201000129.
7. Wessels, L., M. W. Elting, …, K. Weninger. 2007. Rapid membrane fusion of individual virus particles with supported lipid bilayers. *Biophys. J.* 93:526–538. http://www.ncbi.nlm.nih.gov/entrez/query.fcgi?cmd=Retrieve&db=PubMed&dopt=Citation&list_uids=17449662.
8. Floyd, D. L., J. R. Ragains, …, A. M. van Oijen. 2008. Single-particle kinetics of influenza virus membrane fusion. *Proc. Natl. Acad. Sci. USA.* 105:15382–15387.
9. Villamil Giraldo, A. M., S. Mannsverk, and P. M. Kasson. 2022. Measuring single-virus fusion kinetics using an assay for nucleic acid exposure. *Biophys. J.* 121:4467–4475. https://doi.org/10.1016/j.bpj.2022.11.002.
10. Cervantes, M., S. Mannsverk, …, P. M. Kasson. 2025. Single-Virus Microscopy of Biochemical Events in Viral Entry. *JACS Au.* 5:399–407. https://doi.org/10.1021/jacsau.4c00992.
11. Mullner, F. E., S. Syed, …, F. J. Sigworth. 2010. Improved hidden Markov models for molecular motors, part 1: basic theory. *Biophys. J.* 99:3684–3695. https://doi.org/10.1016/j.bpj.2010.09.067.
12. Sgouralis, I., and S. Pressé. 2017. An Introduction to Infinite HMMs for Single-Molecule Data Analysis. *Biophys. J.* 112:2021–2029. https://doi.org/10.1016/j.bpj.2017.04.027.
13. Fox, E. B., E. B. Sudderth, …, A. S. Willsky. 2008. An HDP-HMM for systems with state persistence. *In* Proceedings of the 25th International Conference on Machine Learning ACM, pp. 312–319. https://doi.org/10.1145/1390156.1390196.
14. Syed, S., F. E. Müllner, …, F. J. Sigworth. 2010. Improved hidden Markov models for molecular motors, part 2: extensions and application to experimental data. *Biophys. J.* 99:3696–3703. https://doi.org/10.1016/j.bpj.2010.09.066.
15. Teh, Y. W., M. I. Jordan, …, D. M. Blei. 2006. Hierarchical Dirichlet Processes. *J. Am. Stat. Assoc.* 101:1566–1581. http://www.jstor.org/stable/27639773.
16. Siekmann, I., L. E. Wagner, 2nd, …, J. Sneyd. 2011. MCMC estimation of Markov models for ion channels. *Biophys. J.* 100:1919–1929. https://doi.org/10.1016/j.bpj.2011.02.059.

17. Munro, J. B., J. Gorman, …, W. Mothes. 2014. Conformational dynamics of single HIV-1 envelope trimers on the surface of native virions. *Science.* 346:759–763. https://doi.org/10.1126/science.1254426.

18. Sgouralis, I., S. Madaan, …, S. Pressé. 2019. A Bayesian Nonparametric Approach to Single Molecule Forster Resonance Energy Transfer. *J. Phys. Chem. B.* 123:675–688. https://doi.org/10.1021/acs.jpcb.8b09752.

19. McKinney, S. A., C. Joo, and T. Ha. 2006. Analysis of single-molecule FRET trajectories using hidden Markov modeling. *Biophys. J.* 91:1941–1951. https://doi.org/10.1529/biophysj.106.082487.

20. Sarkar, S. K., B. Marmer, …, K. C. Neuman. 2012. Single-molecule tracking of collagenase on native type I collagen fibrils reveals degradation mechanism. *Curr. Biol.* 22:1047–1056. https://doi.org/10.1016/j.cub.2012.04.012.

21. Ivanovic, T., J. L. Choi, …, S. C. Harrison. 2013. Influenza-virus membrane fusion by cooperative fold-back of stochastically induced hemagglutinin intermediates. *eLife.* 2:e00333. https://doi.org/10.7554/eLife.00333.

22. Ivanovic, T., and S. C. Harrison. 2015. Distinct functional determinants of influenza hemagglutinin-mediated membrane fusion. *eLife.* 4:e11009. https://doi.org/10.7554/eLife.11009.

23. Chao, L. H., D. E. Klein, …, S. C. Harrison. 2014. Sequential conformational rearrangements in flavivirus membrane fusion. *eLife.* 3:e04389. https://doi.org/10.7554/eLife.04389.

24. Rawle, R. J., E. R. Webster, …, S. G. Boxer. 2018. pH dependence of Zika membrane fusion kinetics reveals an off-pathway state. *ACS Cent. Sci.* 4:1503–1510.

25. Weber, T. S., I. Jaehnert, …, J. Carneiro. 2014. Quantifying the length and variance of the eukaryotic cell cycle phases by a stochastic model and dual nucleoside pulse labelling. *PLoS Comput. Biol.* 10:e1003616. https://doi.org/10.1371/journal.pcbi.1003616.

26. Yates, C. A., M. J. Ford, and R. L. Mort. 2017. A Multi-stage Representation of Cell Proliferation as a Markov Process. *Bull. Math. Biol.* 79:2905–2928. https://doi.org/10.1007/s11538-017-0356-4.

27. Shaevitz, J. W., S. M. Block, and M. J. Schnitzer. 2005. Statistical kinetics of macromolecular dynamics. *Biophys. J.* 89:2277–2285. https://doi.org/10.1529/biophysj.105.064295.

28. Schnitzer, M. J., and S. M. Block. 1995. Statistical kinetics of processive enzymes. *Cold Spring Harbor Symp. Quant. Biol.* 60:793–802. https://www.ncbi.nlm.nih.gov/pubmed/8824454.

29. Akaike, H. 1974. A new look at the statistical model identification. *IEEE Trans. Automat. Control.* 19:716–723.

30. Sugiura, N. 1978. Further analysis of the data by akaike's information criterion and the finite corrections: Further analysis of the data by akaike's. *Commun. Stat. Theor. Methods.* 7:13–26.

31. Svoboda, K., P. P. Mitra, and S. M. Block. 1994. Fluctuation analysis of motor protein movement and single enzyme kinetics. *Proc. Natl. Acad. Sci. USA.* 91:11782–11786. https://www.ncbi.nlm.nih.gov/pubmed/7991536.

32. Rawle, R. J., A. M. Villamil Giraldo, …, P. M. Kasson. 2019. Detecting and Controlling Dye Effects in Single-Virus Fusion Experiments. *Biophys. J.* 117:445–452. https://doi.org/10.1016/j.bpj.2019.06.022.

33. Cervantes, M., T. Hess, …, P. M. Kasson. 2023. The ACE-2 receptor accelerates but is not biochemically required for SARS-CoV-2 membrane fusion. *Chem. Sci.* 14:6997–7004. https://doi.org/10.1039/D2SC06967A.

34. Gillespie, D. T. 1976. A general method for numerically simulating the stochastic time evolution of coupled chemical reactions. *J. Comput. Phys.* 22:403–434. https://doi.org/10.1016/0021-9991(76)90041-3.

35. Gillespie, D. T. 2007. Stochastic simulation of chemical kinetics. *Annu. Rev. Phys. Chem.* 58:35–55. https://doi.org/10.1146/annurev.physchem.58.032806.104637.

36. Rawle, R. J., S. G. Boxer, and P. M. Kasson. 2016. Disentangling Viral Membrane Fusion from Receptor Binding Using Synthetic DNA-Lipid Conjugates. *Biophys. J.* 111:123–131. https://doi.org/10.1016/j.bpj.2016.05.048.

37. Schwarz, G. 1978. Estimating the dimension of a model. *Ann. Stat.* 6:461–464.

38. Sengar, A., M. Cervantes, …, P. M. Kasson. 2023. Single-virus fusion measurements reveal multiple mechanistically equivalent pathways for SARS-CoV-2 entry. *J. Virol.* 97:e0199222. https://doi.org/10.1128/jvi.01992-22.

39. Pabis, A., R. J. Rawle, and P. M. Kasson. 2020. Influenza hemagglutinin drives viral entry via two sequential intramembrane mechanisms. *Proc. Natl. Acad. Sci. USA.* 117:7200–7207. https://doi.org/10.1073/pnas.1914188117.

40. Li, T., Z. Li, …, T. Ivanovic. 2021. The shape of pleomorphic virions determines resistance to cell-entry pressure. *Nat. Microbiol.* 6:617–629. https://doi.org/10.1038/s41564-021-00877-0.

41. Floyd, D. L., S. C. Harrison, and A. M. van Oijen. 2010. Analysis of kinetic intermediates in single-particle dwell-time distributions. *Biophys. J.* 99:360–366. https://doi.org/10.1016/j.bpj.2010.04.049.

42. Villamil Giraldo, A. M., and P. M. Kasson. 2020. Bilayer-Coated Nanoparticles Reveal How Influenza Viral Entry Depends on Membrane Deformability but Not Curvature. *J. Phys. Chem. Lett.* 11:7190–7196. https://doi.org/10.1021/acs.jpclett.0c01778.

43. Zhou, P., X. L. Yang, …, Z. L. Shi. 2020. A pneumonia outbreak associated with a new coronavirus of probable bat origin. *Nature.* 579:270–273. https://doi.org/10.1038/s41586-020-2012-7.

44. Hoffmann, M., H. Kleine-Weber, …, S. Pöhlmann. 2020. SARS-CoV-2 Cell Entry Depends on ACE2 and TMPRSS2 and Is Blocked by a Clinically Proven Protease Inhibitor. *Cell.* 181:271–280.e8. https://doi.org/10.1016/j.cell.2020.02.052.

45. Benton, D. J., A. G. Wrobel, …, S. J. Gamblin. 2020. Receptor binding and priming of the spike protein of SARS-CoV-2 for membrane fusion. *Nature.* 588:327–330. https://doi.org/10.1038/s41586-020-2772-0.

46. Koch, J., Z. M. Uckeley, …, P.-Y. Lozach. 2021. TMPRSS2 expression dictates the entry route used by SARS-CoV-2 to infect host cells. *EMBO J.* 40:e107821. https://doi.org/10.15252/embj.2021107821.

47. Lu, M., P. D. Uchil, …, W. Mothes. 2020. Real-time conformational dynamics of SARS-CoV-2 spikes on virus particles. *Cell Host Microbe.* 28:880–891.e8.

48. Yoshina-Ishii, C., Y. H. M. Chan, …, S. G. Boxer. 2006. Diffusive dynamics of vesicles tethered to a fluid supported bilayer by single-particle tracking. *Langmuir.* 22:5682–5689. http://www.ncbi.nlm.nih.gov/entrez/query.fcgi?cmd=Retrieve&db=PubMed&dopt=Citation&list_uids=16768494.

49. Danieli, T., S. L. Pelletier, …, J. M. White. 1996. Membrane fusion mediated by the influenza virus hemagglutinin requires the concerted action of at least three hemagglutinin trimers. *J. Cell Biol.* 133:559–569. http://www.ncbi.nlm.nih.gov/entrez/query.fcgi?cmd=Retrieve&db=PubMed&dopt=Citation&list_uids=8636231.

50. Ellens, H., J. Bentz, …, J. M. White. 1990. Fusion of influenza hemagglutinin-expressing fibroblasts with glycophorin-bearing liposomes: role of hemagglutinin surface density. *Biochemistry.* 29:9697–9707. http://www.ncbi.nlm.nih.gov/entrez/query.fcgi?cmd=Retrieve&db=PubMed&dopt=Citation&list_uids=2271610.

51. Morris, S. J., D. P. Sarkar, …, R. Blumenthal. 1989. Kinetics of pH-dependent fusion between 3T3 fibroblasts expressing influenza hemagglutinin and red blood cells. Measurement by dequenching of fluorescence. *J. Biol. Chem.* 264:3972–3978. http://www.ncbi.nlm.nih.gov/entrez/query.fcgi?cmd=Retrieve&db=PubMed&dopt=Citation&list_uids=2917985.

52. Otterstrom, J. J., B. Brandenburg, …, A. M. van Oijen. 2014. Relating influenza virus membrane fusion kinetics to stoichiometry of neutralizing antibodies at the single-particle level. *Proc. Natl. Acad. Sci. USA.* 111:E5143–E5148. https://doi.org/10.1073/pnas.1411755111.

53. Mannsverk, S., A. M. Villamil Giraldo, and P. M. Kasson. 2025. Influenza viral infection at the plasma membrane is restricted by

lipid composition. *J. Virol.* 99:e0110525. https://doi.org/10.1128/jvi.01105-25.

54. Carr, C. M., C. Chaudhry, and P. S. Kim. 1997. Influenza hemagglutinin is spring-loaded by a metastable native conformation. *Proc. Natl. Acad. Sci. USA.* 94:14306–14313. http://www.ncbi.nlm.nih.gov/entrez/query.fcgi?cmd=Retrieve&db=PubMed&dopt=Citation&list_uids=9405608.
55. Shang, J., G. Ye, …, F. Li. 2020. Structural basis of receptor recognition by SARS-CoV-2. *Nature.* 581:221–224. https://doi.org/10.1038/s41586-020-2179-y.
56. Yan, R., Y. Zhang, …, Q. Zhou. 2020. Structural basis for the recognition of SARS-CoV-2 by full-length human ACE2. *Science.* 367:1444–1448. https://doi.org/10.1126/science.abb2762.
57. Wrapp, D., N. Wang, …, J. S. McLellan. 2020. Cryo-EM structure of the 2019-nCoV spike in the prefusion conformation. *Science.* 367:1260–1263. https://doi.org/10.1126/science.abb2507.
58. Bel, G., B. Munsky, and I. Nemenman. 2009. The simplicity of completion time distributions for common complex biochemical processes. *Phys. Biol.* 7:016003. https://doi.org/10.1088/1478-3975/7/1/016003.

Biophysical Journal, Volume *124*

Supplemental information

Unmasking complex kinetics in viral entry by inferring hypoexponential models

Oyinkansola Adenekan and Peter M. Kasson

Supplementary Information for

Unmasking complex kinetics in viral entry by inferring hypoexponential models

Oyinkansola Adenekan[1] and Peter M. Kasson[1,2,3*]

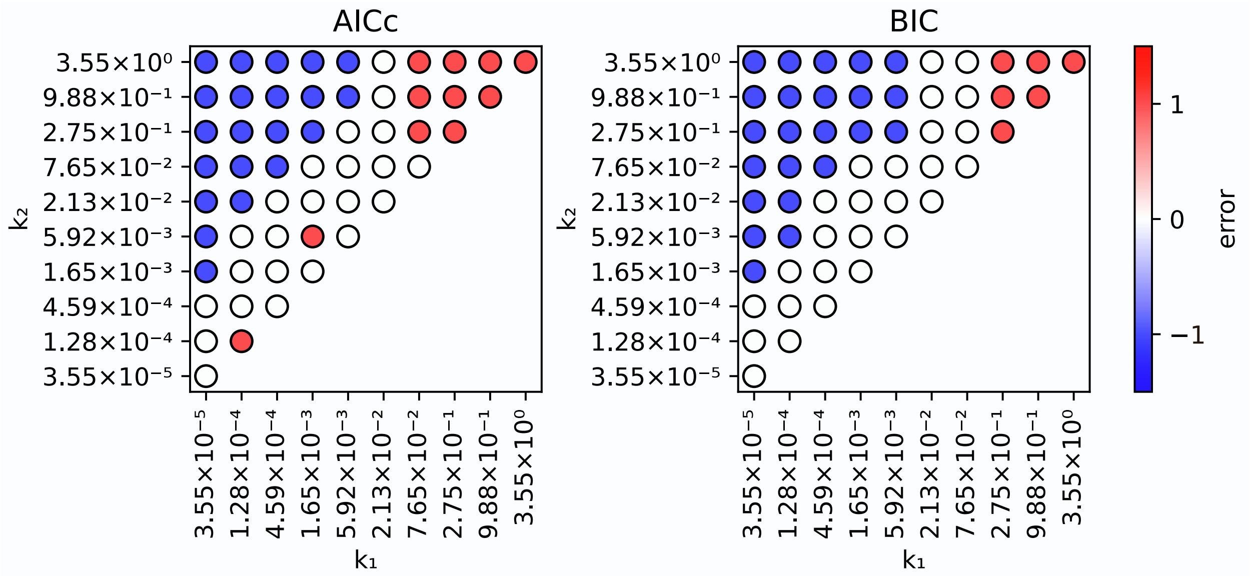

Figure S1. Model selection using AICc versus Bayesian Information Criterion (BIC). The estimated number of steps N was selected using AICc or BIC on hypoexponential sampling of models with 1-5 steps. This was performed on three independent sampling runs, and the majority-vote selection was adopted. This is analogous to the AICc model selection presented in Figure 3c.

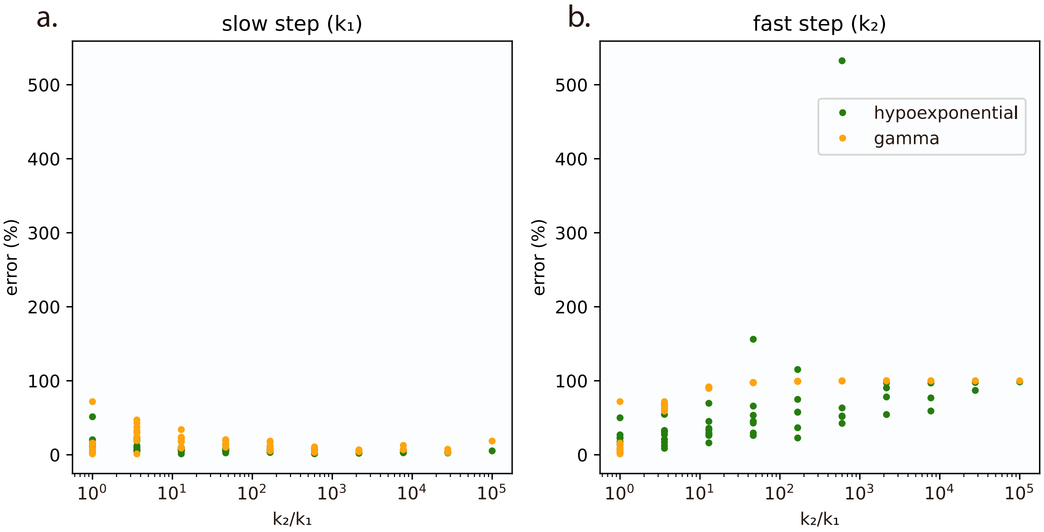

Figure S2. Rate errors as a function of k_2/k_1 ratio. Data from Figure 3 are replotted as a function of k_2/k_1 ratio in panel (a) for k_1 and panel (b) for k_2.

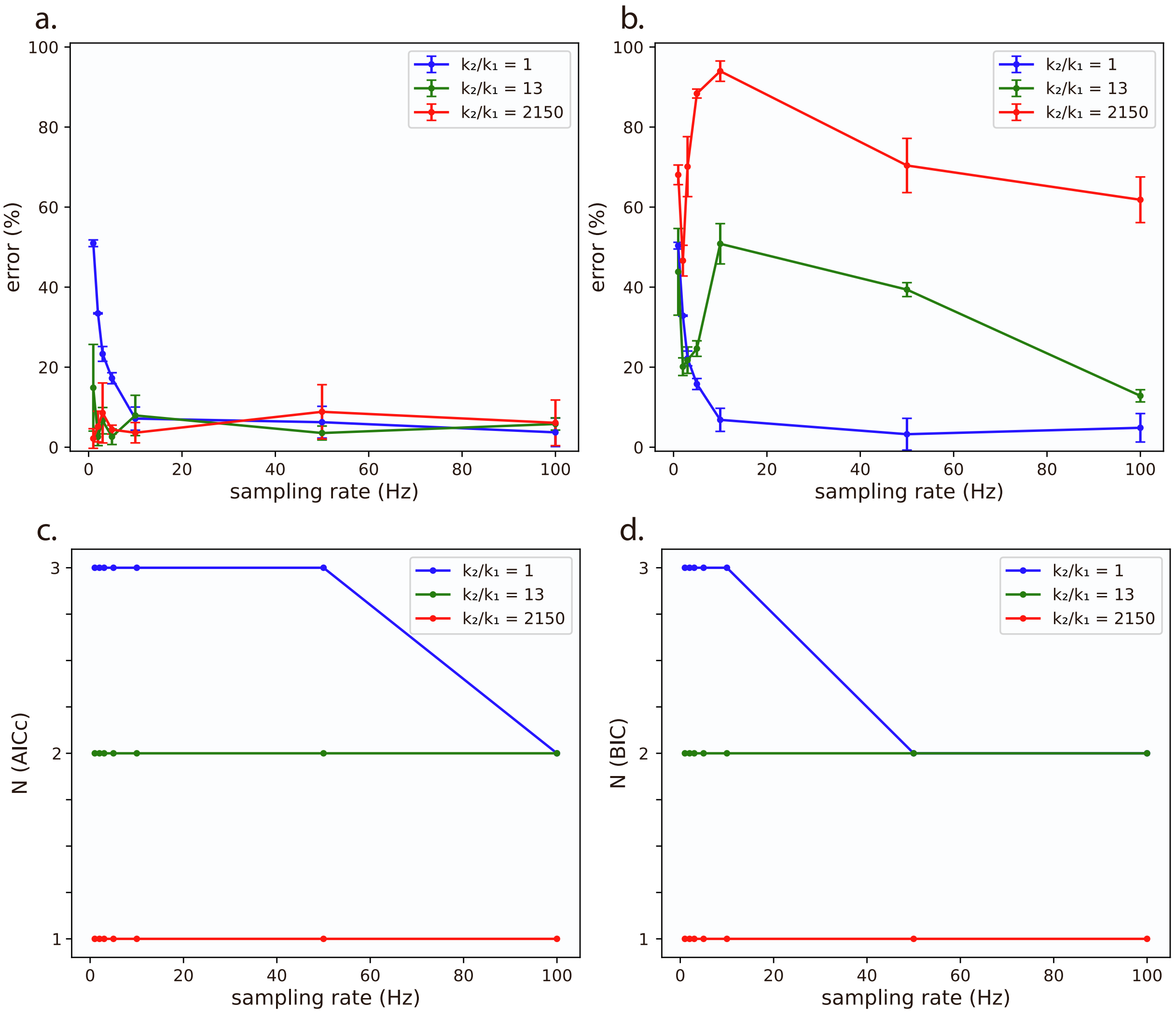

Figure S3. Dependence of error on sampling rate. Synthetic data were generated to emulate experimental sampling rates or time resolutions varying from 1 Hz to 100 Hz, and the error was calculated for three different two-step rate regimes, k_1 = 3.55 s^{-1} and k_2 = 3.55 s^{-1} (blue), k_1 = 2.13e-02 s^{-1} and k_2 = 2.75e-01 s^{-1} (green), and k_1 = 4.59e-04 s^{-1} and k2 = 9.88e-01 s^{-1} (red). The errors were plotted in panel (a) for k_1, panel (b) for k_2, panel (c) for N using AICc model selection, and panel (d) for N using BIC model selection. In all cases, increasing the sampling rate reduced error for faster rates. Three separate runs of hypoexponential sampling were performed, and error bars in panels a-b show standard deviation. Panels c and d show errors based on majority-vote.

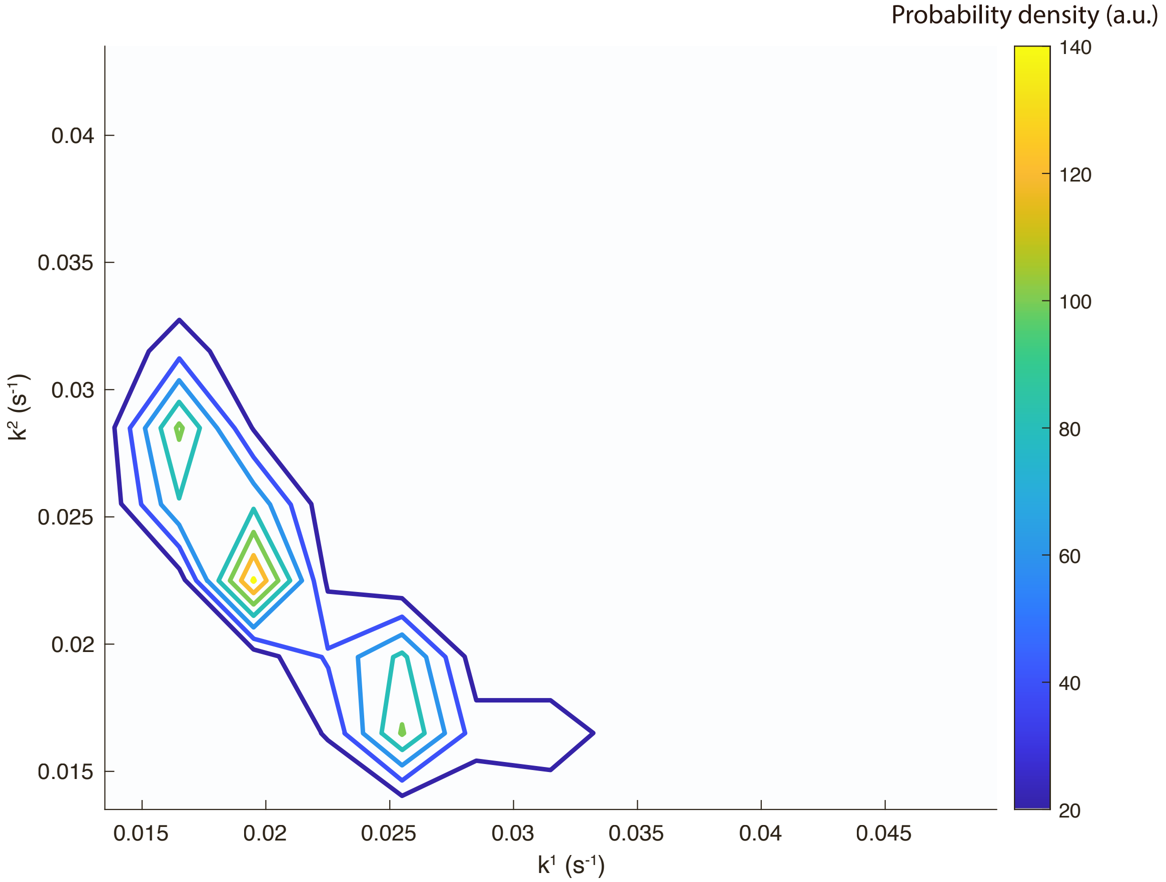

Figure S4. Joint distribution of estimated rates for influenza fusion. Lipid-mixing kinetics from influenza virus fusion were sampled using hypoexponential fits. The joint distribution of k_1 and k_2 is plotted here as a two-dimensional contour plot.

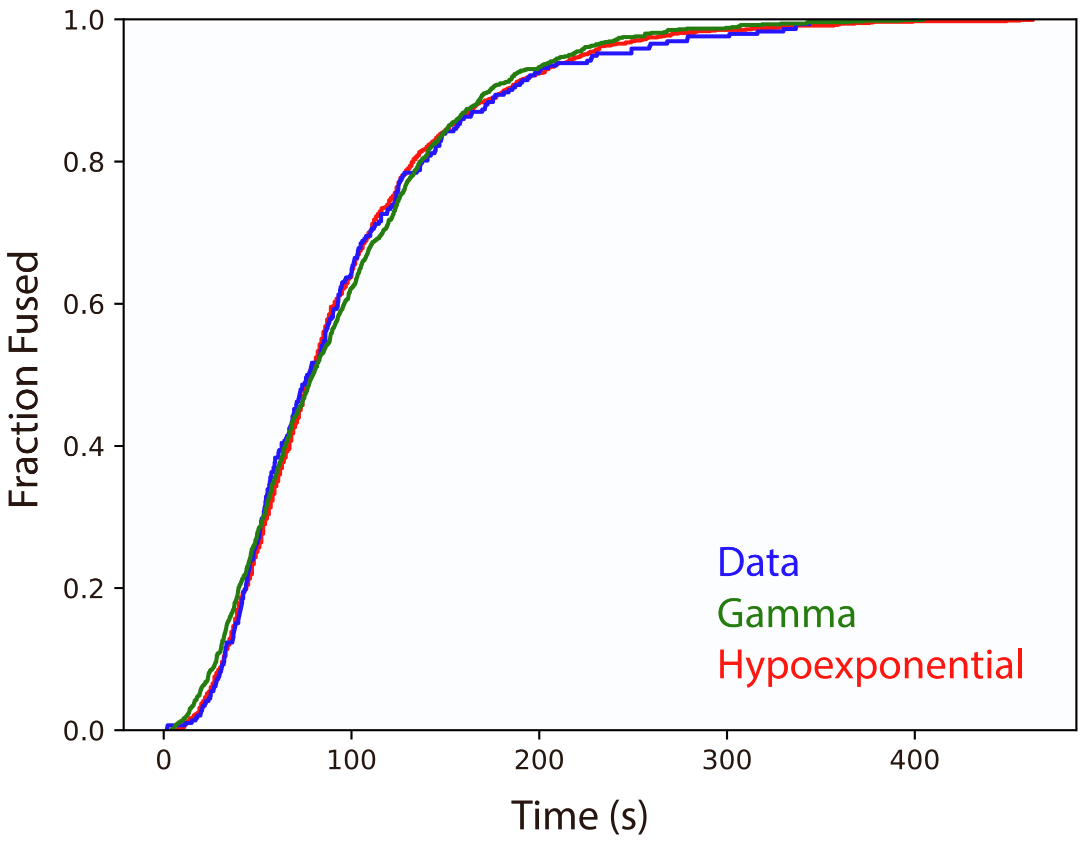

Figure S5. Comparison of gamma and hypoexponential CDFs for influenza lipid mixing kinetics. Simulated dwell times were used to reconstruct CDFs for each fitting method and plotted with the experimental data. Both methods well match the observed kinetics.

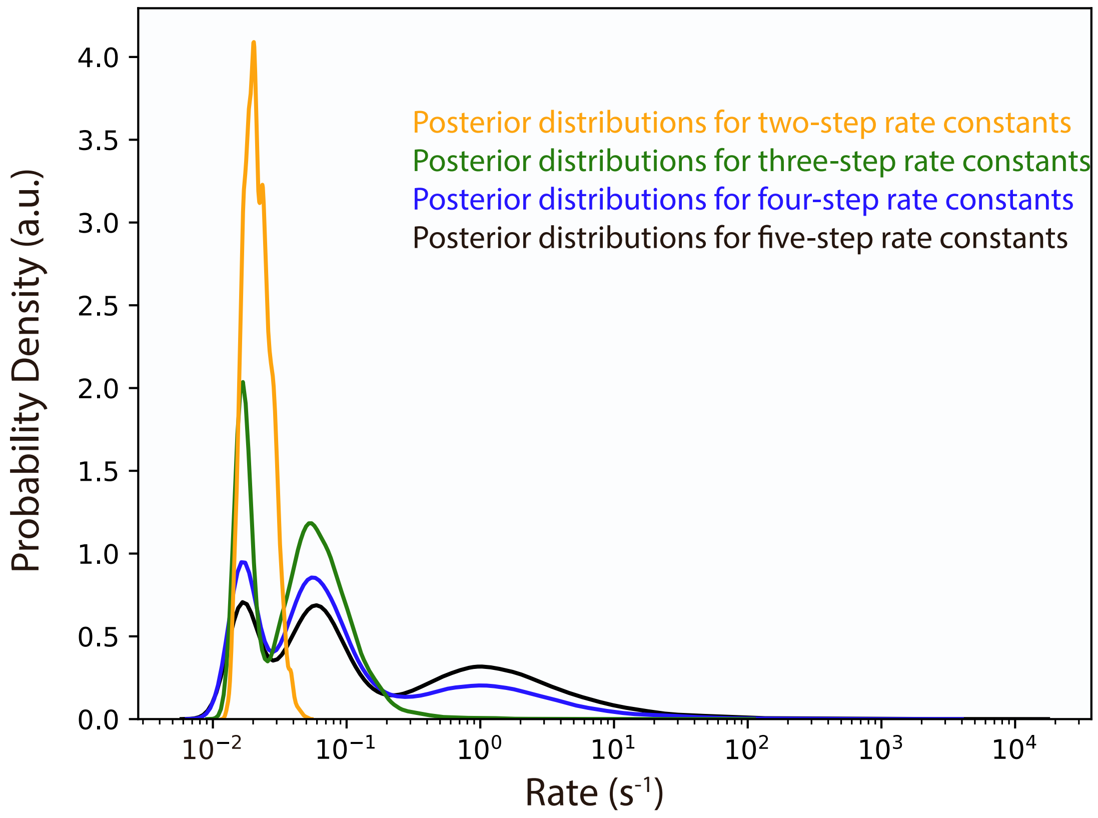

Figure S6. Posterior distributions for rate constants corresponding to 2, 3, and 4 step models for influenza fusion. Rate constants were sampled using 10,000 MCMC steps. Two-step rate constants have an essentially unimodal distribution, while three, four, and five show progressive splitting of the modes towards faster rate constants.

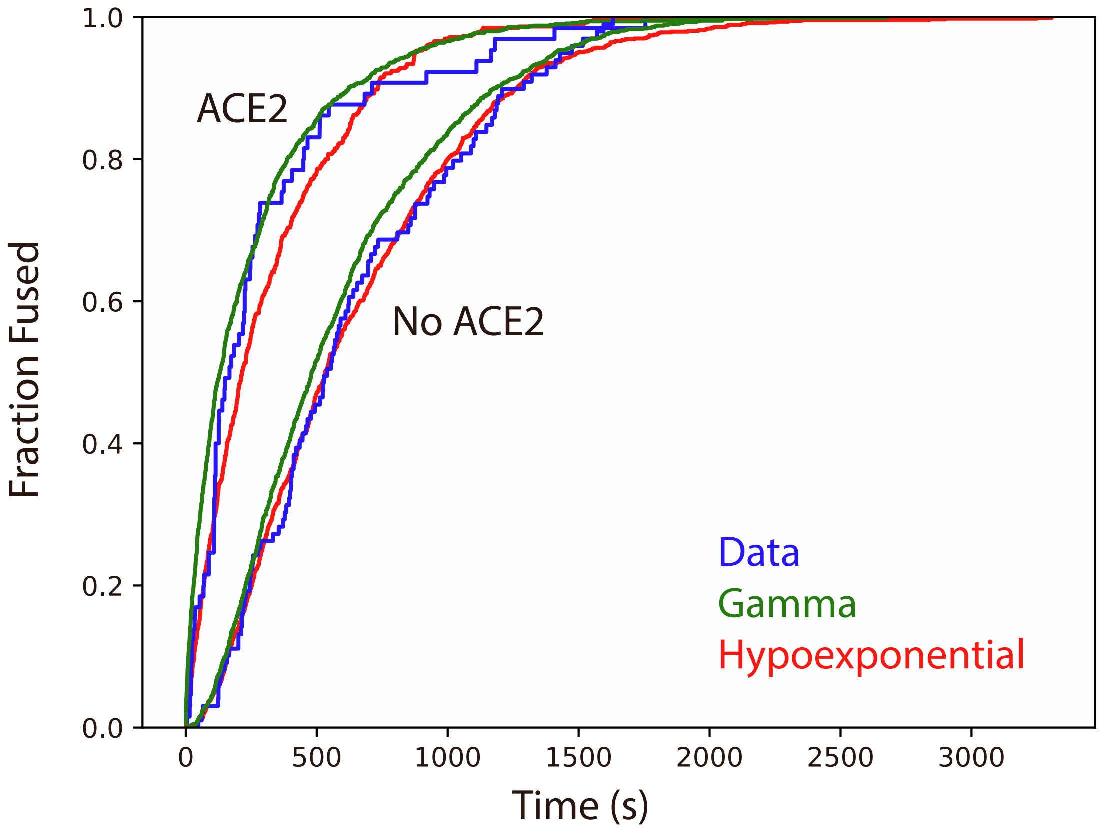

Figure S7. Comparison of gamma and hypoexponential CDFs for SARS-CoV-2 lipid mixing kinetics. Simulated dwell times were used to reconstruct CDFs for each fitting method and plotted with the experimental data. Hypoexponential distributions fit slightly better for fusion in the absence of ACE2, while gamma distributions fit slightly better for fusion in the presence of ACE2 (perhaps due to non-integer N values).

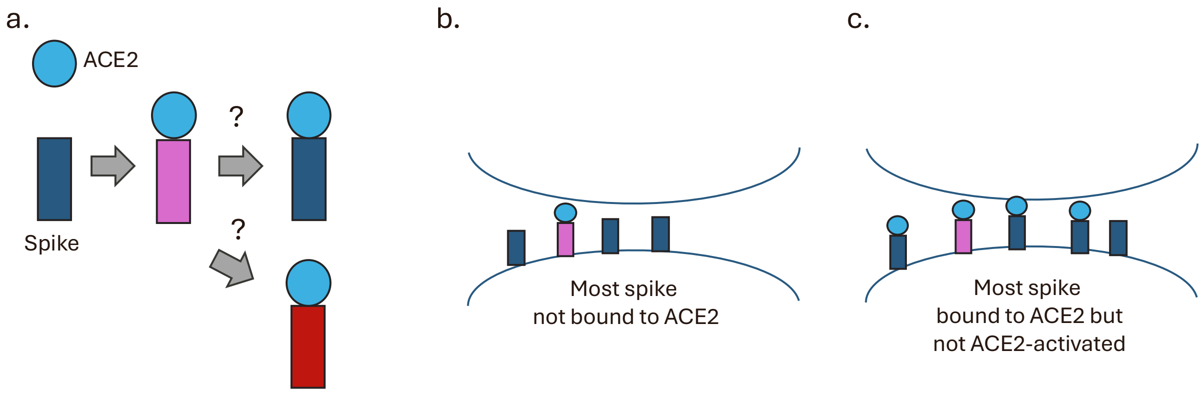

Figure S8. Schema of mixed activation scenarios. Schematized in panel (a) is the activation and inactivation of spike glycoprotein by ACE2. Activated spike is denoted in magenta. It is not known whether activated spike inactivates to baseline (teal) or to a completely unreactive spike (red). Two potential scenarios where the asymmetric model for fusion would apply are schematized in panels (b) and (c). In (b), most spike is not ACE2-bound. This is likely not true at the concentrations of soluble ACE2 used in our experiments. In (c), most spike is ACE2-bound but inactive. This could result either in mixed activation by bound, activated spike and unbound spike or, if inactive ACE2-bound spike retains baseline activity, mixed activation by bound, activated spike and bound, inactivate spike.
